# Antioxidative and Anticancer Potential of Luteolin: A Comprehensive Approach Against Wide Range of Human Malignancies

**DOI:** 10.1002/fsn3.4682

**Published:** 2025-01-19

**Authors:** Mahwish, Muhammad Imran, Hammad Naeem, Muzzamal Hussain, Suliman A. Alsagaby, Waleed Al Abdulmonem, Ahmed Mujtaba, Mohamed A. Abdelgawad, Mohammed M. Ghoneim, Ahmed H. El‐Ghorab, Samy Selim, Soad K. Al Jaouni, Ehab M. Mostafa, Tadesse Fenta Yehuala

**Affiliations:** ^1^ Institute of Food Science and Nutrition University of Sargodha Sargodha Pakistan; ^2^ Department of Food Science and Technology University of Narowal Narowal Pakistan; ^3^ Department of Food Science and Technology Muhammad Nawaz Shareef University of Agriculture Multan Pakistan; ^4^ Department of Food Sciences Government College University Faisalabad Faisalabad Pakistan; ^5^ Department of Medical Laboratory Sciences, College of Applied Medical Sciences Majmaah University AL‐Majmaah Saudi Arabia; ^6^ Department of Pathology, College of Medicine Qassim University Buraidah Saudi Arabia; ^7^ Department of Food Sciences and Technology, Faculty of Engineering and Technology Hamdard University Islamabad campus Islamabad Pakistan; ^8^ Department of Pharmaceutical Chemistry, College of Pharmacy Jouf University Aljouf Saudi Arabia; ^9^ Department of Pharmacy Practice, College of Pharmacy AlMaarefa University Riyadh Saudi Arabia; ^10^ Department of Chemistry, College of Science Jouf University Sakaka Saudi Arabia; ^11^ Department of Clinical Laboratory Sciences, College of Applied Medical Sciences Jouf University Sakaka Saudi Arabia; ^12^ Department of Hematology/Oncology, Yousef Abdulatif Jameel Scientific Chair of Prophetic Medicine Application, Faculty of Medicine King Abdulaziz University Jeddah Saudi Arabia; ^13^ Department of Pharmacognosy, College of Pharmacy Jouf University Sakaka Saudi Arabia; ^14^ Pharmacognosy and Medicinal Plants Department, Faculty of Pharmacy (Boys) Al‐Azhar University Cairo Egypt; ^15^ Faculty of Chemical and Food Engineering, Bahir Dar Institute of Technology Bahir Dar University Bahir Dar Ethiopia

**Keywords:** anticancer, antidiabetic, anti‐inflammatory, antioxidant, flavonoid, luteolin, phytochemical

## Abstract

Luteolin is widely distributed phytochemical, a flavonoid, in kingdom plantae. Luteolin with potential antioxidant activity prevent ROS‐induced damages and reduce oxidative stress which is mainly responsible in pathogenesis of many diseases. Several chemo preventive activities and therapeutic benefits are associated with luteolin. Luteolin prevents cancer via modulation of numerous pathways, that is, by inactivating proteins; such as procaspase‐9, CDC2 and cyclin B or upregulation of caspase‐9 and caspase‐3, cytochrome C, cyclin A, CDK2, and APAF‐1, in turn inducing cell cycle arrest as well as apoptosis. It also enhances phosphorylation of p53 and expression level of p53‐targeted downstream gene. By Increasing BAX protein expression; decreasing VEGF and Bcl‐2 expression it can initiate cell cycle arrest and apoptosis. Luteolin can stimulate mitochondrial‐modulated functions to cause cellular death. It can also reduce expression levels of p‐Akt, p‐EGFR, p‐Erk1/2, and p‐STAT3. Luteolin plays positive role against cardiovascular disorders by improving cardiac function, decreasing the release of inflammatory cytokines and cardiac enzymes, prevention of cardiac fibrosis and hypertrophy; enhances level of CTGF, TGFβ1, ANP, Nox2, Nox4 gene expressions. Meanwhile suppresses TGFβ1 expression and phosphorylation of JNK. Luteolin helps fight diabetes via inhibition of alpha‐glucosidase and ChE activity. It can reduce activity levels of catalase, superoxide dismutase, and GS4. It can improve blood glucose, insulin, HOMA‐IR, and HbA1c levels. This review is an attempt to elaborate molecular targets of luteolin and its role in modulating irregularities in cellular pathways to overcome severe outcomes during diseases including cancer, cardiovascular disorders, diabetes, obesity, inflammation, Alzheimer's disease, Parkinson's disease, hepatic disorders, renal disorders, brain injury, and asthma. As luteolin has enormous therapeutic benefits, it could be a potential candidate in future drug development strategies.

## Graphical abstract

Luteolin prevents cancer via targeting numerous cellular components, signaling/inflammatory pathways and transcription factors that result in initiation of cell cycle arrest, apoptosis, autophagy, and metastasis. Luteolin also provides protection against cardiovascular disorders, diabetes, obesity, inflammation, Alzheimer's disease, Parkinson's disease, hepatic disorders, renal disorders, brain injury, and asthma by targeting specific molecules, inflammatory factors, and modulation of irregularities in cellular pathways.

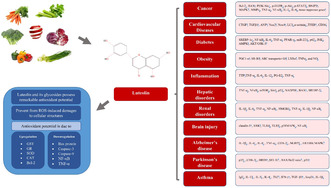

## 1 Introduction

**FIGURE 1:**
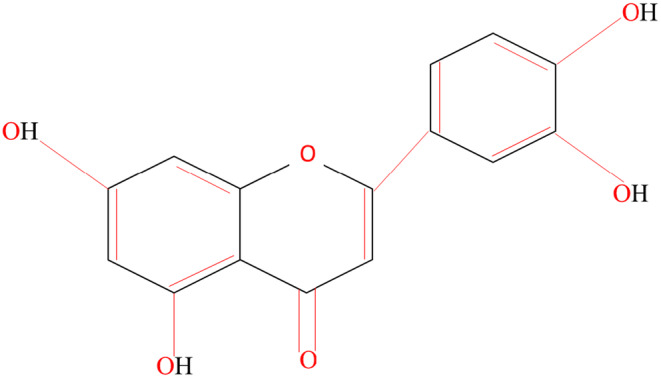
Chemical structure of luteolin.

Luteolin (3′, 4′, 5, 7‐tetrahydroxyflavone) is a secondary metabolite of many plant, belongs to flavone subgroup of flavonoids. Structurally, it possesses one oxygen‐containing ring, two benzene rings, and four hydroxyl (–OH) groups (Figure 1). Its biological and biochemical activities are mainly due to presence of OH group and 2–3 double bonds (Imran et al. 2019). Luteolin with molecular formula of C_15_H_10_O_6_ has molecular weight of 286.24 and weakly soluble in aqueous solution ( Shakeel et al. 2018). In plants, luteolin may be present as aglycone or glycoside form, that is, without or with a sugar molecule attached to it, respectively. The sugar molecules mostly bound to luteolin includes glucose, arabinose, xylose, rutinose, rhamnose, glucuronic acid, and some other sugar derivatives (Wang, Wuniqiemu, et al. 2021; Wang, Zeng, et al. 2021). In general, luteolin glycosides are O‐glycosides in which one or more freely reactive OH group in the sugar molecule is attached to the aglycone at positions 3′, 4′, 5 and 7. Some examples directly belong to the O‐glycosides are cynaroside (luteolin 7‐O‐ß‐d‐glucoside) and scolymoside (luteolin 7‐O‐rutinoside). Sugar molecules also attach to luteolin by carbon–carbon linkage and produce C‐glycoside, which includes orientin and isoorientin. These luteolin O‐ and C‐glycosides are particularly important in food supplements and pharmaceutical industries due to comparatively less cytotoxic effects.

**FIGURE 2:**
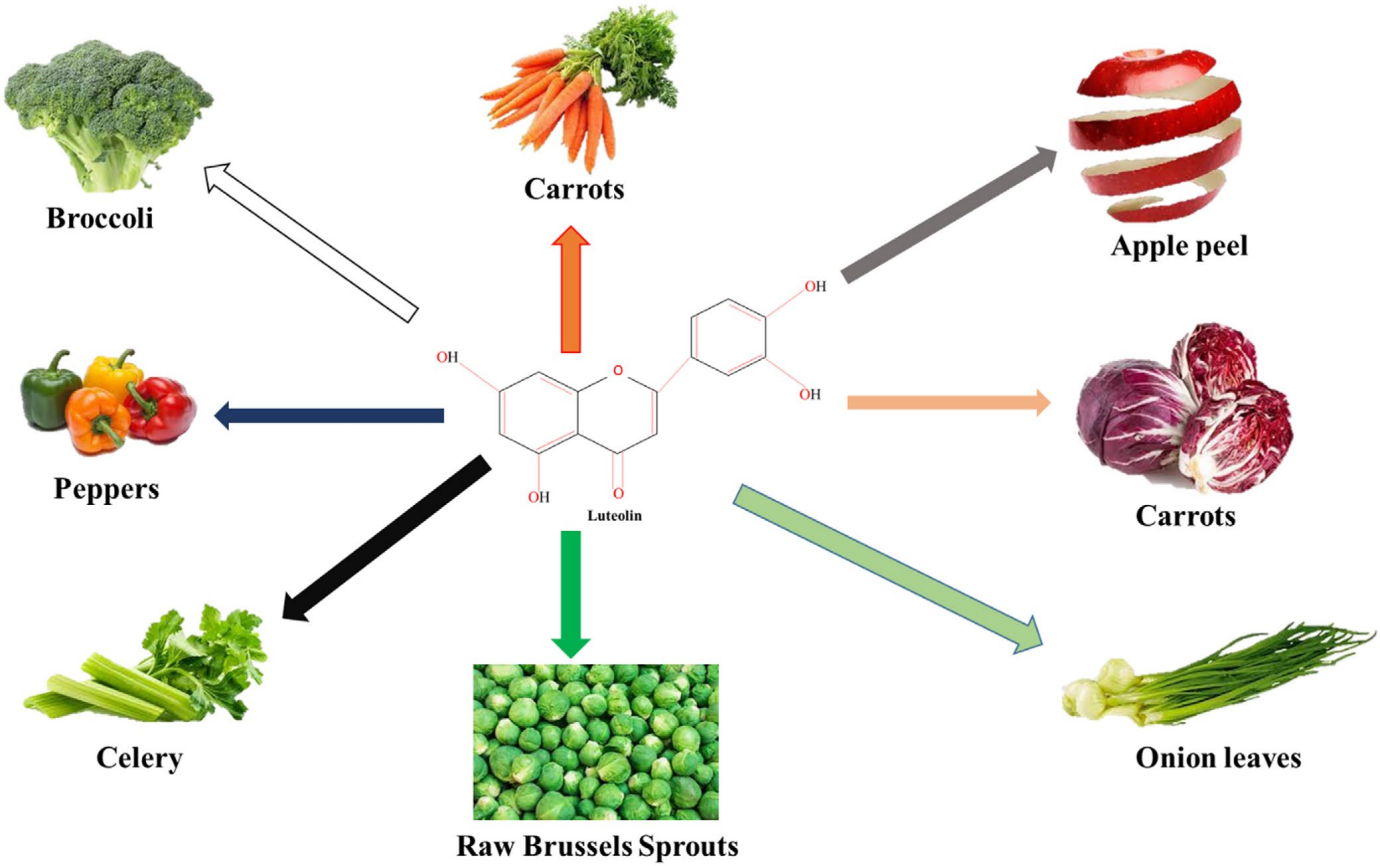
Sources of luteolin.

The luteolin has wide distribution in many plant families as more than 300 species were documented to possess luteolin and its glycosides (López‐Lázaro 2009). Luteolin was found even in Ulmus and Celtis which were 25‐ and 36‐million years old fossil species (Giannasi and Niklas 1977). The edible sources of luteolin includes peppers, onion, peppermint, carrots, turnip, cauliflower, cabbage, spinach, rosemary, celery, thyme, fennel, Perilla leaves, Brussels sprouts, buckwheat sprouts, chives, harwort, oregano, artichoke, pomegranate, skin of apple, lemon, lettuce, chocolate, parsley, capers, cucumber, horseradish, beets, olive oil, rooibos tea, and green tea (López‐Lázaro 2009; Caporali et al. 2022). Natural sources (fruits and vegetables) of luteolin were shown in Figure 2.

In plants, the biosynthesis of luteolin starts with precursor amino acid phenylalanine which is converted into trans‐cinnamic acid by an enzymatic reaction using phenylalanine ammonia lyase as catalyst followed by formation of trans‐coumaric acid, p‐coumaroyl CoA, naringenin chalcone, and naringenin using enzymes trans‐cinnamate 4‐hydroxylase, coumarate 4‐ligase, chalcone synthase, chalcone isomerase, respectively (Martens and Mithöfer 2005; Ferrer et al. 2008). Furthermore, naringenin is directly converted into luteolin using flavone synthase enzyme. In addition, naringenin may convert into apigenin by enzymatic activity of flavone synthase enzyme or eriodictyol by the enzyme flavonoid 3′‐hydroxylase. These apigenin and eriodictyol molecules may also be converted into luteolin with the help of enzymes flavonoid 3′‐hydroxylase and flavone synthase, respectively (Nabavi et al. 2020).

The bioavailability of luteolin is very low, although it is abundantly present in dietary sources. Due to the momentous first pass effect, only 4.10% was found to be available from dosage of 50 mg/kg intake of luteolin (Sarawek, Derendorf, and Butterweck 2008). A very small amount of free luteolin is present in the body after intestinal absorption as most of luteolin is converted into conjugated molecules. It was observed that luteolin, while passing through the intestinal mucosa, converted into glucuronides. Hence, luteolin in free form as well as methylated and other conjugated forms were found in human plasma. Furthermore, the HPLC analysis confirmed that human sera contained luteolin in free state and monoglucuronide form after its ingestion (Shimoi et al. 1998).

In another study, luteolin was administered orally to rats and it was observed that luteolin glucuronides were present in various organs and in plasma (Hayasaka et al. 2018). It was also detected that luteolin monoglucuronide, luteolin monoglucoside, and luteolin were present in free form (Yasuda et al. 2015). In a study, the maximum plasma concentration was found to be 1.97 ± 0.15 g/mL and half‐life of luteolin were found as 4.94 ± 1.2 h after administration of 14.3 mg/kg dose orally to experimental rats (López‐Lázaro 2009). The presence of luteolin in free state might be due to the escape of some luteolin from the hepatic methylation/sulfation or intestinal conjugation. In an experiment, luteolin and its active metabolites were analyzed by using HPLC‐MS/MS in rat plasma which indicated that pharmacological activities or toxicity of this molecule is due to the activity of UDP‐glucuronosyltransferase enzymes (Shi et al. 2018). Hence, the luteolin bioactivity is mainly due to its metabolites (Kure et al. 2016), enzymatic activities of UDP‐glucuronosyltransferases and in certain circumstances due to catalytic activity of catechol‐O‐methyltransferases (Wang et al. 2017). In addition, less bioavailability and resultantly limited clinical applications of this molecule is due to enzymatic activity of uridine diphosphate glucuronosyltransferases 1As (UGT1As) which cause excessive glucuronidation of luteolin. Recently, it was reported that the activity of UGT1A1 and UGT1A9 were inhibited by potent inhibitors like resveratrol which substantially increase luteolin bioavailability by suppressing the amount of glucuronidation metabolites (Wu et al. 2022). In addition, the solubility and bioactivity of luteolin was found to improve by glycosylation. Some other methods like nanoparticles encapsulation and formation of luteolin‐loaded self‐emulsified phospholipid preconcentrate enhance the absorptivity and bioactivity of luteolin (Hsieh et al. 2022).

In traditional Chinese medicine system, luteolin enriched plants have been utilized to treat various ailments such as hypertension, inflammatory disorders, obesity, diabetes, and cancer (Harborne and Williams 2000). Several chemopreventive activities are considered to be associated with luteolin such as antioxidative, anticancer, antitumor, anti‐inflammatory, antidiabetic, apoptotic, autophagic‐regulatory, antimicrobial, and neuroprotective functions (Luo et al. 2019; Ma et al. 2019; Yu et al. 2019; Li, Wang, et al. 2023).

Luteolin and its derivatives are diverse and have potential to be used in the prevention and control of many maladies. The mechanisms behind luteolin health benefits are scarcely available. Hence, the present review is planned to elaborate molecular targets of luteolin and its role in modulating irregularities in cellular pathways to overcome severe outcomes during disease progression.

## 2 Antioxidant Potential

The structure of luteolin classically exhibits antioxidant features due to double covalent bonding between C2 and C3 in conjugation with C4 linkage to oxo group which is crucial to bind transition metal ions (TMI) like copper or iron and the presence of catechol group in B‐ring which serves as hydrogen or electron donor for the stabilization of radical species (Rice‐Evans, Miller, and Paganga 1996; Mira et al. 2002). The plants containing luteolin and its glycosides possess remarkable antioxidant potential (López‐Lázaro 2009; Tian et al. 2021). The antioxidant potential of luteolin and its glycosides is mainly due to scavenging activity against reactive oxygen species (ROS) and nitrogen species (Ahmadi et al. 2020; Ozgen, Kilinc, and Selamoğlu 2016). ROS is a diverse group of oxygen‐containing species which are highly reactive and short‐lived such as singlet oxygen (^1^O_2_), hydrogen peroxide (H_2_O_2_), hydroxyl radical (OH), lipid peroxyl radical (LOO•), peroxynitrite (ONOO^−^), and superoxide ($O_{2}^{\cdot -}$). For cellular signaling, the ROS acts as second messenger (Tian et al. 2021). However, production of ROS in excessive amounts may generate oxidative stress which has detrimental effects on DNA, proteins and lipids. The luteolin has potential to prevent these ROS‐induced damages to these cellular structures (Alshehri et al. 2020; Manzoor et al. 2019). Oxidative stress is mainly responsible in pathogenesis of many diseases such as diabetes, hypertension, cardiovascular diseases, cancer, neurodegenerative diseases, ischemia/reperfusion (I/R) injury, and aging (Ganai et al. 2021; Kang et al. 2017). Luteolin as an antioxidant molecule has preventive role against oxidative stress and resultant chemotoxicity. Luteolin decreases oxidative stress against HgCl_2_‐induced liver injury by suppressing nuclear factor‐κB (NF‐κB), tumor necrosis factor‐α (TNF‐α), and P53 protein levels (Zhang, Xing, et al. 2017). In H_2_O_2_‐treated cells, cellular lipids and DNA components were damaged due to increased level of Intracellular ROS which were significantly reduced by luteolin pretreatment. Luteolin decreases levels of Bax protein, increases the expression of Bcl‐2, suppresses the active caspase‐3, ‐9, and restores the levels of glutathione as well as upregulates other antioxidant enzymes (Fernando et al. 2024).

In addition, several enzymes that are involved in catalytic oxidation of cellular components are directly inhibited by luteolin. An important marker to determine liver lipids damages is malondialdehyde and its formation in liver lipids is actively suppressed by luteolin through limiting the enzymatic activities of cyclooxygenase and lipoxygenase as well as nonenzymatic oxidation of lipids (Dong et al. 2017).

Luteolin may also have a role in protection and enhancement of endogenous antioxidants such as glutathione‐S‐transferase (GST), glutathione reductase (GR), superoxide dismutase (SOD), and catalase (CAT) (Manju and Nalini 2005; Leung et al. 2006). Moreover, luteolin may chelate TMI that are particularly involved in generating ROS and inducing oxidative damages through the Fenton reaction (Mira et al. 2002) and retards the lipooxygenase activity and control the nontransition metal‐dependent oxidation (Ross and Kasum 2002). Luteolin is also essential in inhibition of pro‐oxidant enzymes activity (Hu and Kitts 2004) and stimulation of certain antioxidant enzymes (Lim et al. 2007; Choi et al. 2008). Luteolin supplementation significantly suppressed the lipid peroxidation and restored the antioxidant enzymes level in several in vivo and in vitro studies (Manju, Balasubramaniyan, and Nalini 2005). As a strong antioxidant molecule, luteolin oral supplementation significantly lowered the concentrations of lipid peroxidation and OH− concentration and increased the antioxidant enzymes level in plasma and colonic mucosa (Ashokkumar and Sudhandiran 2008). In a recent study, it was noticed that 3′, 4′‐dihydroxy arrangement of luteolin glucosides possess a key role for superior antioxidant potential of luteolin (Xu et al. 2022).

## 3 Health Perspectives

### 3.1 Anticancer Potential of Luteolin

Luteolin is a potent molecule that effectively inhibits invasion, migration, and proliferation of cancerous cells and alleviates inflammation and can be a potential candidate in the novel anticancer drugs development (Table 1). Different mechanisms such as autophagy or apoptosis are involved by which luteolin advocates the death of malignant cells. Luteolin possesses pleiotropic antineoplastic activity as it has potential to induce cell cycle arrest, inhibit proliferation of cancer cells, angiogenesis, and metastasis capacity, and induce apoptosis in cancerous experimental subjects (Cai et al. 2011; Ding et al. 2014).

**TABLE 1:** Health perspectives and basic mechanisms of action of luteolin.

Maladies	Mechanism	Reference
Cancer	Inactivate proteins such as procaspase‐9, CDC2, cyclin B; upregulate caspase‐9 and‐3, cytochrome C, cyclin A, CDK2, and APAF‐1 to induce cell cycle arrest and apoptosis	Chen, Tien, et al. (2018)
	Retards proliferation; blocks s‐phase of cell cycle; inhibits PI3K/Akt signaling pathway to induce apoptosis	Yajie et al. (2023)
	Limit expression levels of p‐Akt, p‐EGFR, p‐Erk1/2, and p‐STAT3; inhibit CDK2 activity	Imran et al. (2019)
	Suppress cell proliferation, migration, and invasion; support cellular apoptosis by downregulation of SGK1 and AKT3 and upregulation of BNIP3 gene	Wu et al. (2023)
	Different molecular subtypes of breast cancer cells showed some common and some different effects	Garcia‐Guasch et al. (2023)
	Reduce cell viability in p53‐deficient cell lines	Jang et al. (2019)
	Initiate cell cycle arrest and apoptosis by enhancing phosphorylation of p53 and expression level of p53‐targeted downstream gene	Yoo, Won, and Kwon (2021)
	Arrest G2/M phase that initiate cellular apoptosis	Chen, Tien, et al. (2018)
	Increase BAX protein expression; decrease VEGF and Bcl‐2 expression; stimulate mitochondrial‐modulated functions to cause cellular death	Sabzichi et al. (2014)
	Suppress hTERT in MDA‐MB‐231 breast cancer cell line; modify NF‐κB inhibitor α phosphorylation; target c‐Myc gene to regulate cell cycle	Huang, Jin, and Lan (2019)
	Activate CARD; stimulates caspase‐9 and other caspase cascade reactions to initiate cellular apoptosis	Wang et al. (2019); Lee, Park, Kang, et al. (2021); Lee, Park, Bae, et al. (2021)
	Induce apoptosis by suppressing tumor proliferation after activation of MAPK pathway	Zhang et al. (2021)
	Control expression of LIM domain kinase 1, p‐cofilin, p‐LIMK, and Ki‐67 proteins	Zhang et al. (2021)
	Boost DR5 expression which activate caspase‐3, caspase‐8, and caspase‐9	Park et al. (2014)
	Increases the expression of caspase‐1, IL‐1β, Gasdermin D, and induce pyroptosis	Chen et al. (2022)
	Activate beclin 1 and caspases that increase the autophagy flux and MAP 1A/1B‐light chain 3 (LC3) puncta	Park et al. (2013); Lee, Park, Kang, et al. (2021); Lee, Park, Bae, et al. (2021)
	Decrease Akt phosphorylation; triggers GSK‐3 and blocks the Wnt β‐catenin pathway; activate GSK‐3 to phosphorylate cyclin D1 that destruct proteasomes	Uekita et al. (2013)
	Downregulate P62 to initiates autophagy	Potočnjak et al. (2020)
	Downregulate ANO1 expression	Seo et al. (2017)
	Enhance calcified nodules; promote cell viability and ALP activity; enhance runt‐related transcription factor 2, osteocalcin, bone morphogenetic protein 2, Osterix, and β‐catenin and cyclin D1 expressions	Quan et al. (2019)

	Block TNF‐α, NF‐κB, IL‐1, IL‐6, and proinflammatory cytokines as well as endothelial migratory expressions of MMPs	Fang et al. (2018); Kang et al. (2017); Li, Chen, et al. (2017); Li, Lin, et al. (2017)
	Suppress tumor‐associated macrophages; hinder release of chemokines	Fang et al. (2018); Kang et al. (2017)
	Suppress EMT essentially notable in cancer metastasis	Hussain et al. (2021)
	Alter the mTOR, ERK, PI3K/AKT, p38 signaling pathways, and activities of related molecules	Lin et al. (2022)
	Block EGFR‐signaling pathway and FAK activity	Kim et al. (2013); Li, Chen, et al. (2017); Li, Lin, et al. (2017)
	Increase cell invasion and loss of cellular adhesion through AKT pathway via increasing E‐cadherin expression by inhibiting mdm2	Zhou et al. (2009)
	Regulate expression of genes related to tumor formation	Mishan et al. (2021)
	Enhance miR‐34a and miR‐16 expression; reduce miR‐21 expression	Magura, Moodley, and Mackraj (2022)
	Inhibit NOTCH signaling hyperactivation by upregulation of miR‐34a, miR‐121a, miR‐139‐5p, miR‐246, miR‐224, and downregulation of miR‐155	Sun et al. (2015)
	Reduce cellular growth; downregulate miR 301 to trigger apoptosis; induce production of DED‐containing protein 2	Han et al. (2016)
	Target MDM4 and increasing miR‐34a‐5p expression to stop carcinogenesis	Jiang et al. (2018)
	Reduce cellular proliferation; boost TRAIL induced antiproliferative effects by downregulating miR‐301‐3p	Moeng et al. (2020)
	Modulate PTN via altering the miR‐384 expression	Yao et al. (2019)
	Activate MAPK to inhibit tumor growth by triggering autophagy and apoptosis in glioma cells	Yao et al. (2019)
	Induce apoptosis, phosphorylation of JNK, suppression of NF‐κB translocation	Hu et al. (2012)
	Suppress mdm2 levels; downregulate TGF‐β1 and E‐cadherin as well as physiological appearance of cancer cells	Zhou et al. (2009); Chen et al. (2013)
	Reduce cell viability, proliferation, invasion, and colony formation of GICs and tumor organoids	Yi et al. (2018)
	Suppress cell proliferation, invasion, migration, induction of apoptosis, reversion of EMT, downregulation of vimentin, N‐cadherin, Snail, and induction of E‐cadherin expressions	Zang et al. (2017)
	Prevent proliferation of PC‐3 cells via suppressing Wnt signaling pathways through upregulating the transcriptional FZD6, and inhibiting the stemness of prostrate cancer cells	Han et al. (2018)
	Reduce PTTG1 oncoprotein overexpression	Chen, Tien, et al. (2018)
	Downregulate pleiotrophin expression; upregulate miR‐384 expression	Yao et al. (2019)
Repress Zta and Rta genes as well as Matrigel invasiveness; reduce genomic instability and tumorigenicity	Wu et al. (2016)

	Lower the tumor dimension, inhibit cell proliferation and mTOR signaling, lower the Ki67‐labeling index and p‐S6 expressions	Iida et al. (2020)
	Influence cellular migration, invasion and lowered the p38 phosphorylation and MMP‐2 expression	Velmurugan et al. (2020)
	Inhibits lung cancer cells migration by upregulating miR‐106a‐5p via negatively regulated MMP2 and TWIST1	Wang et al. (2024)
	Inhibit matrix metalloproteinase‐9 expressions; induced apoptosis via extrinsic and intrinsic pathways	Lee et al. (2019)
	Diminish the activity of phosphorylated nonreceptor tyrosine kinase, Ras homolog gene family member A, Ras‐related C3 botulinum toxin substrate 1, phosphorylated focal adhesion kinase, and CDC protein 42 expressions	Masraksa et al. (2020)
	Binds directly with KDM4C; epigenetically inhibits PPP2CA/YAP axis that results in decreased stemness of ovarian cells	Li, Wang, et al. (2023); Li, Li, et al. (2023)
	Increase cytokine production, cell chemotaxis, and T‐cell activation; luteolin maintain high ratio of CD8+ T lymphocytes	Cai et al. (2024)
	Inhibits tumor resistance; enhanced drug chemosensitivity by inhibiting PI3K/AKT signaling pathway	Yang et al. (2024)
	Modulate gut microbiota, reduce number of tumors in case of colorectal cancer	Pérez‐Valero et al. (2024)
The nanoparticles encapsulation of luteolin is more effective in inhibition of PC3 and HeLa cells from proliferation and migration in prostate and anticervical cancer experiments	Ding et al. (2024)
Cardiovascular disorders	Enhance antioxidative, antiapoptotic, anti‐inflammatory properties	Pan et al. (2022)
	Improve cardiac function; lower active caspases‐3 and ‐9; decrease the proapoptotic protein Bax; increase peroxiredoxin II expressions	Wei et al. (2018)
	Improve cardiac function, decrease in release of inflammatory cytokines and cardiac enzyme after MI	Hu et al. (2016)
	Prevent from cardiac fibrosis and hypertrophy; enhance level of CTGF, TGFβ1, ANP, Nox2, Nox4 gene expressions Suppress TGFβ1 expression and phosphorylation of JNK	Nakayama et al. (2015)
	Suppress stimulated noncollagen extracellular matrix genes and collagen genes; prevent from myocardial injury and fibrosis; block ISO‐stimulated expression of Rac2, Ncf1, Ncf4, Cyba, and Cybb genes and revert ISO‐altered expression of microRNAs	Ning et al. (2017)
	Attenuate inflammatory responses, alleviate mitochondrial injury, reduce oxidative stress, inhibit cardiac apoptosis, enhance autophagy, improve cardiac functions, and reduce phosphorylation of AMPK	Wu et al. (2020)
	Ameliorate cardiac activity, increase sumoylation of SRCA2a; enhance phosphorylation of PLB, Akt; improve contractility and Ca2+ transients, suppress PI3K/Akt pathway, improve myocardium fibrosis; upregulate expression ratio of Bcl‐2/Bax, caspase‐3/cleaved‐Caspase3	Hu et al. (2017)
	Decrease the expression of α‐actinin and LC3; increased cell viability of cardiomyocytes
Li et al. (2019)

	Inhibit phlpp1 activity primarily needed in upregulation of AKT/Bcl‐2 pathway	Zhang et al. (2020)
	Lower the lactate dehydrogenase release, myocardial infarct size, and apoptosis	Nai et al. (2015)
	Reduce apoptosis regulator Bax expression; enhance Bcl‐2 expression; lower the concentration of urea, creatinine, MDA and lactate dehydrogenase; suppress L‐type calcium currents	Yan et al. (2019)
	Induce apoptosis, reactive oxygen development, promotion of mitochondrial autophagy, loss of mitochondrial membrane potential; facilitate phosphorylation of Drp1 at Ser616; upregulate TFEB expression	Xu et al. (2020)
	Minimize the production of ROS, enhance HO‐1 and Nrf2 levels; reduce the oxidative damages and inflammation	Li et al. (2019)
	Reduce cardiomyocyte apoptosis, attenuate myocardial injury, enhance SERCA2a transcriptional activity; improve left ventricular function by upregulating Sp1 expression; increase cardiomyocytes contractile capacity	Hu et al. (2020)
	Control progression of atherosclerosis via suppressing STAT3	Ding et al. (2019)
Reduces hyperlipidemia‐induced cardiac damages by Suppressing lipid deposition (LOX‐1 and CD36) and cardiac fibrosis (MMP2, MMP9, collagen I, collagen III, and TGF‐β)	Dong et al. (2023)
Diabetes	Inhibit alpha‐glucosidase activity	Djeujo et al. (2022)
	Improve blood glucose, insulin, HOMA‐IR, and HbA1c levels	Zang, Igarashi, and Li (2016)
	Minimize catalase, superoxide dismutase, and GS4 activity in hippocampus and cerebral cortex of rats; inhibit ChE activity	Liu et al. (2013)
	Potentiate insulin signaling; enhance glucose metabolism; regulate β‐cell function; increase hepatic insulin sensitivity	Park et al. (2018)
	Enhances insulin secretion, improves β‐cell dysfunction, reduces inflammation via modulation of NF‐kB, IL‐6, TNF‐α, and PPAR‐γ expressions and downregulation of SREBP‐1c	Shehnaz et al. (2023)
	Improves β‐cell dysfunction, insulin resistance, endothelial function; Inhibits the activities of α‐glucosidase and dipeptidyl peptidase‐4	Chang and Yue (2024)
	Trigger sestrin 2‐Nrf2‐based feedback loop	Zhou et al. (2021)
	Induce antioxidants in diabetic nephropathy; imparts renoprotective effects by enhancing the HO‐1 expression; prevention kidney from morphological damages during diabetes	Wang, Wuniqiemu, et al. (2021); Wang, Zeng, et al. (2021)
	Initiate insulin action; boost PPAR‐γ target genes expression	Ding, Jin, and Chen (2010)
	Improves insulin secretion; decrease Maft activator through NF‐κB signaling pathway	Ding et al. (2014)

	Provide protection against diabetic cardiomyopathy by down regulation of miR‐221, p62,JNK, and c‐Jun, reduction in mitochondrial swelling, enhancing autophagic vesicles and LC3‐II/I in the diabetic heart	Xiao et al. (2023)
Protects from diabetic cardiomyopathy by reducing proteasome activity and upregulating AMPK and AKT/GSK‐3 pathways	Zhang et al. (2023)
Obesity	Increase metabolomic rates; reduce diet‐induced obesity by activating the AMPK/PGC1α pathway	Zhang et al. (2016)
	Reduce cholesterol by regulating cholesterol efflux genes	Park et al. (2020)
	Decrease infiltration of macrophages and dysregulation of adipokine/cytokine; modify Toll‐like receptor signaling pathways	Kwon et al. (2018b)
	Regulate activities against adipocyte differentiation by modulating the TF PPAR‐γ	Park et al. (2009)
	Reduce obesity adipocyte inflammation by blocking activity of proinflammatory mediators in macrophages such as monocyte chemoattractant protein, TNF‐α, and NO	Ando et al. (2009)
	Manage body weight, lipid and glycemic parameters, and physiological aspects related to hepato‐ and cardiometabolic parameters	Terzo et al. (2023)
	Enhances adipose tissue thermogenesis, increases systemic energy expenditure, inhibit lipogenesis and inflammation in adipose tissues	Zhang et al. (2024)
Inflammation	Inhibit the activity of proinflammatory cytokines; relieves chronic pharyngitis from inflammation via blocking polarization of M1 macrophage and NF‐κB pathway	Chen, Tien, et al. (2018)
	Reduce number of inflammatory cells, NO, TNF‐α, and prostaglandin (PG) E2 levels	Kanai et al. (2016)
	Suppress inflammatory cytokines; reduce the histamine release	Jeon et al. (2014)
	Block ERK, MEK, and Akt phosphorylation	Lee et al. (2010)
	Inhibit the proinflammatory markers expression; block proapoptotic genes expression	Dirscherl et al. (2010)
	Blockade of Src kinase and Syk kinase; inhibition of PGE2, NO and, nuclear translocation of NF‐κB (p50 and p65); suppression of TNF‐α, COX‐2, and iNOS mRNA transcripts levels	Lee et al. (2015)
	Alleviates oxidative stress and inflammation via inhibition of NOX4‐mediated NF‐κB signaling pathway and levels of inflammation factors	Li, Wang, et al. (2023); Li, Li, et al. (2023)
	Inhibit TNF‐α‐induced adhesion of monocytes, TNF‐α‐induced expressions of adhesion molecules; inhibit NF‐κB transcriptional activity, nuclear translocation of NF‐κB p65, and expression of IKKß	Jia et al. (2015)
	Inhibit PPAR‐γ/STAT/MyD88 pathway	Miao, Li, and Li (2018)
Liver disorders	Reduce serum ALT, AST, TNF‐α, cyclooxygenase levels and its transcription factors, activator protein (AP)‐1, and NF‐κB, decreasing the Phase II enzymes levels and activation of Nrf‐2	Park and Song (2019)
	Provide protection against hepatic injury by modulating cytochrome P450, oxidative phosphorylation, and certain signaling pathways	Qu et al. (2020)
	Maintain concentration of AST, ALT, and total bilirubin, increase antioxidant enzymes, reduce NO, MDA, iNOS, TNF‐α, IL‐1β, and nuclear factor kappa B level; upregulate Bcl‐2 and reduce BAX and caspase‐3 proteins	AL‐Megrin et al. (2019)
	Improves liver lesions by minimizing unnecessary accumulation of extracellular matrix, reducing oxidative stress, inhibiting inflammatory markers, and regulating lipid balance	Yao et al. (2023)
	Reduce hepatotoxicity by decreasing TNF‐α, NF‐κB, mTOR, Sirt1, p53; downregulate p38 MAPK activation	Yang et al. (2016)
	Reduce oxidative stress in liver by modulating associated factors and pathways	Zhang, Xing, et al. (2017); Zhang, Tan, et al. (2017); Castellino et al. (2019)
Kidney disorders	Decrease the concentrations of MDA, MPO, 8‐oxo‐deoxyguanosine, urea nitrogen and creatinine level; enhance antioxidant enzymes; attenuate the increased expression levels of IL‐1β, IL‐6, TNF‐α, NF‐κB and (HMGB1); significantly reduce renal cell apoptosis and ER stress	Hong et al. (2017)
	Reduce TNF‐α, NF‐κB, IL‐1β, cleaved caspase‐3, MCP‐1, and ICAM‐1 expression in LPS‐induced renal injury mice	Xin et al. (2016)
	Restore cellular viability of damaged renal tissue	Liu et al. (2017)
	Protect against renal failure through the detoxification mechanism	Albarakati et al. (2020)
	Reduce hepatoxicity via boosting antioxidant enzymes activity	Yan et al. (2019)
	Luteolin reduced renal injury by inhibiting XO activity, modulating uric acid transporters, as well as activating Nrf2 HO‐1/NQO1 antioxidant pathways and renal SIRT1/6 cascade. KIM‐1, caspase‐3, and renal ATP‐binding cassette subfamily G isoform 2 protein expressions were reduced	Yu et al. (2024)
Brain injury	Induce autophagy for neuroprotection; ameliorate secondary brain injuries	Xu et al. 2014
	Decrease oxidative damage and neuroinflammation; activate microglia; improve neurotrophic potential to protect dopaminergic neurons	Patil et al. (2014)
	Protect brain from ischemic damage by reducing oxidative stress and apoptosis; upregulate claudin‐5 expressions	Qiao, Zhang, et al. (2012)
	Protect brain from the damage caused by MCA by upregulating ERK expression and downregulating TLR4, TLR5, p38MAPK, and NF‐κB	Qiao, Dong, et al. (2012)
	Inhibit the production of neuronal mitochondrial superoxides	Tan et al. (2019)
Alzheimer's disease	Increase cell viability of hAs and hBMECs after fAβ_1–40_ injury; preserve transendothelial electrical resistance to protect barrier function; reduce fAβ_1–40_‐induced cytokine production and inflammatory mediator, including IL‐1β, IL‐6, IL‐8, TNF‐α, and COX‐2; regulate inflammatory signal transduction	Zhang, Xing, et al. (2017); Zhang, Tan, et al. (2017)
	Reduce AchE activity to slow down inception of Alzheimer's disease‐like symptoms	Ali et al. (2019)
	Reduce proinflammatory proteins NLRP3, and transcriptional expression of acute‐phase protein IL‐1β, TNF‐α, SAA1, IFN‐γ, and CB2 receptors and TCR‐ζ, CD3‐γ, CD137, Fpr2, TLR2 chain	Contarini et al. (2019)
	Reverse mitochondrial membrane potential dissipation, decrease the Aβ1‐42 secretion, down regulate the AβPP level, and increase the SOD activity	Liu et al. (2011)
	Decrease protein plaques, improve histomorphology of brain; inhibit astrocyte hyperactivation and neuroinflammation; reduce memory deficits and improve spatial learning	Kou et al. (2021)
	Increase doublecortin‐immunoreactive cells, bromodeoxyuridine‐positive nuclei; enhance expression of the nerve growth factor, brain‐derived neurotrophic factor, glial cell‐derived neurotrophic factor, and neurotrophin‐3	Crupi et al. (2016)
	Inhibit Aβ25‐35; induce cellular apoptosis; activate ER/ERK/MAPK pathways; produce apoptotic bodies via direct stimulation of ERβ	Wang et al. (2020)
	Reduce apoptotic cell death, oxidative stress, neuroinflammation, synaptic dysfunction, and amyloid production by inhibition of JNK	Ahmad et al. (2021)
	Activates mTORC1 and attenuates glutamate‐induced autophagy‐mediated cell death	Vongthip et al. (2024)
	Binds directly with PPAR‐γ to enhance its expression and functional aspects; repair mitochondrial damages; reduces neuronal apoptosis; inhibits Aβ generation	He et al. (2023)
Parkinson's disease	Potent antioxidative and anti‐inflammatory activity; influence multilayer modulatory pathways	Goyal, Solanki, and Verma (2024)
	Suppress p35 and cyclin‐dependent kinase‐5 expressions	Reudhabibadh et al. (2021)
	Protect against dopaminergic neuro injury by suppressing apoptosis; reduce caspase‐3 to increase Bcl2/BAX ratio; increase neuro fibers in striatum and restore TH positive neurons in the substantia nigra	Qin et al. (2019)
	Modulate PD associated genes, oxidative stress responses, and cellular pathways related to inflammation in microglial cell line BV2; protect from rotenone toxicity	Elmazoglu et al. (2020)
	Inhibit 6‐OHDA‐induced apoptosis; block BIM and TRB3 mRNA expression	Hu et al. (2014); Guo et al. (2012)
	Suppress ER stress by increasing mRNA levels of HRD1 and SEL1L and their protein expressions	Nishiguchi et al. (2024)
Asthma	Inhibit the mucus overproduction as well as suppress the GABAA receptors	Shen et al. (2016)
	Stimulate CD4 + CD25 + foxp3 + Treg cells to manage asthma	Kim et al. (2018)
	Reduce serum CD4 + IL‐4‐secreting T cells, HDM‐specific IgE, allergic symptoms, infiltration of eosinophils, mucus secretion, expression of GATA3 and pSTAT6, percentage of CD4 + IL‐4‐secreting splenocytes expression	Liang et al. (2020)
	Inhibit human ANO1 activity; stop increase in electrolyte transport induced by ATP	Kim et al. (2020)
	Decrease inflammation in allergic asthma by activating Treg cells	Seumois et al. (2020); Lan et al. (2020)
	Inhibit autophagy in pulmonary tissues via restricting beclin1‐PI3KC3 protein complex and activating PI3K/Akt/mTOR signaling pathway	Wang, Wuniqiemu, et al. (2021); Wang, Zeng, et al. (2021)
	Reduce M1 polarization and enhance M2 polarization of macrophages by stimulation of THP‐1; mediate USP4 and hsa‐miR‐136‐5p expressions	Gong et al. (2022)
	Inhibits IL‐36γ secretion‐mediated MAPK pathways to alleviate asthma	Qiao et al. (2023)

During apoptosis, multiple pathways are implicated such as modulation of mitochondrial membrane, activation of caspase‐3, ‐7, ‐8, and ‐9, release of cytochrome c, suppression of antiapoptotic proteins such as levels of B cell leukemia/lymphoma 2 (Bcl‐2) and Bcl‐xL, increment of death receptors, downregulation of certain factors including TNF receptor, DR4, DR5, Fas/FasL, and apoptosis‐inducing ligand (TRAIL) (Ham et al. 2014). After receiving signals of apoptosis, binding of Fas‐associated death domain (FADD) starts which results in the formation of initiator caspases‐8 and ‐10 due to continuous recruitment of death‐induced signaling complex (Park et al. 2013). Many intrinsic and extrinsic apoptotic pathways are also activated because of mitochondrial membrane disruption which triggers caspase activities, promotes imbalance in the Bcl‐2‐associated X (BAX)/Bcl‐xL ratio, and lowers the proteins expressions of survivin, p21, mdm2, and Mcl‐1 (Lim et al. 2007; Chen et al. 2012; Rao, Ellerby, and Bredesen 2004). After luteolin treatment, inhibition of cell proliferation, reduction in antiapoptotic protein Bcl‐2, increase in BAX levels, and induction of apoptosis in BEL‐7402 and SMMC‐7721 cell lines were observed (Ding et al. 2014).

Cell cycle arrest and induction of apoptosis are regarded as fundamental approaches in the treatment of cancer. Luteolin was found to have a role in inducing apoptosis in cancer cells via extrinsic and intrinsic mechanisms. Luteolin arrests the cell cycle at different phases that have resulted in initiation of apoptosis in cancer cell lines (Chen, Tien, et al. 2018). In gastric cancer cells, luteolin retards proliferation and blocks s‐phase of cell cycle. The inhibition of PI3K/Akt signaling pathway in these cells was the fundamental approach in induction of apoptosis (Yajie et al. 2023). This apoptotic induction is mainly due to inactivation of proteins essentially required for accomplishment of different phases of cell cycle such as procaspase‐9, cell division control (CDC) 2 and cyclin B and upregulation of cyclin‐dependent kinases (CDK) 2, caspase‐9 and ‐3, cytochrome C, cyclin A, and APAF‐1 (Chen, Tien, et al. 2018). The role of these proteins is essential in cell cycle progression and during division of cells. Moreover, luteolin effectively limits the expression levels of many proteins including extracellular signal‐regulated kinase (phosphorylated (p)‐Erk1/2), epidermal growth factor receptors, (p‐EGFR and p‐Akt), and activator of transcription and signal transducer (p‐STAT3) in cancer cells (Imran et al. 2019). In triple‐negative breast cancer, luteolin suppress cell proliferation, migration, and invasion while supporting cellular apoptosis dose‐ and time‐dependently by downregulation of SGK1 and AKT3 and upregulation of BNIP3 gene (Wu et al. 2023). Different molecular subtypes of breast cancer cells showed some common effects after luteolin treatment (formate overflow, acetate production, and stimulation of mitochondrial metabolism) while modulated differentially with respect to lipids and glucose metabolism (Garcia‐Guasch et al. 2023).

In p53‐deficient cell lines, luteolin administration (25 μM) substantially reduces cell viability due to induction of apoptosis and simultaneously significant increase in the cell proportion was observed at G0/G1‐phase and momentous reduction was observed at S‐phase of cell cycle (Jang et al. 2019). Luteolin enhances the phosphorylation of p53 and expression of p53‐targeted downstream genes that initiate cell cycle arrest and ultimately apoptosis (Yoo, Won, and Kwon 2021). In OCM‐1 and HT‐29 cancerous cells, the inhibition of CDK2 activity is responsible for cell cycle arrest at G1 (Imran et al. 2019). Moreover, dose‐ and time‐dependent effects were observed after luteolin treatment in the human colon LoVo and HCT‐15 cell lines (Turktekin et al. 2011). In addition, cell cycle arrest was observed at G2/M phase due to luteolin that resultantly initiate cellular apoptosis (Chen, Tien, et al. 2018). Luteolin cause death of cancer cells as it increases BAX protein expression as well as significantly reduces Bcl‐2 and vascular endothelial growth factor (VEGF) expression and these signals resultantly stimulated mitochondrial‐modulated functions to cause cellular death (Sabzichi et al. 2014). The activation of caspase recruitment domain (CARD) is due to activity of apoptotic protease activating factor1 (APAF1) and cytochrome C which ultimately stimulates caspase‐9 that initiate formation of apoptotic bodies. Meanwhile, other caspase cascade reactions specifically caspase‐3 reactions initiate those results in cellular apoptosis (Sun et al. 2012). In human glioblastoma cell lines (U‐373MG and A172) and esophageal cancer cell line (Eca109), similar effects were also observed in the course of luteolin‐induced apoptosis (Wang et al. 2019; Lee, Park, Kang, et al. 2021).

Recently, it was studied that suppression of tumor proliferation by luteolin is due to stimulation of mitogen‐activated protein kinase (MAPK) pathway which induce apoptosis. It was also noted that some proteins such as p‐LIMK, p‐cofilin, LIM domain kinase 1 protein, and Ki‐67 that have high expression level in lung cancerous cell lines are substantially controlled by luteolin (Zhang et al. 2021). In lung cancer cells, luteolin triggered death of cancerous cells and also stopped carcinogenesis by targeting MDM4 and increasing miR‐34a‐5p expression (Jiang et al. 2018). According to Hu and coworkers, the antiproliferative and anticancer role of luteolin in human nonsmall‐cell lung cancer A549 cells has been found to be due to induction of apoptosis, phosphorylation of JNK, and suppression of NF‐κB translocation. A549 lung cancer cells lines were prevented from proliferation via down regulating TGF‐β1 and E‐cadherin as well as physiological appearance of these cells was maintained after luteolin treatment (Chen et al. 2013). In A549 lung cancer and MRC‐5 noncancer cell lines, luteolin dose rate of 40 μM did not show any cytotoxic activity whereas different doses of luteolin at 20–40 μM markedly inhibited the cancer stages in dose dependent fashion such as diminished the activity of phosphorylated nonreceptor tyrosine kinase, Ras homolog gene family member A, Ras‐related C3 botulinum toxin substrate 1, phosphorylated focal adhesion kinase, and CDC protein 42 expressions (Masraksa et al. 2020).

In MCF‐7 breast cancer cell line, luteolin induces cell cycle arrest and apoptosis via boosting death receptors DR5 expression, which activates caspase‐3, caspase‐8, and caspase‐9 (Park et al. 2014). Furthermore, luteolin increases the expression of caspase‐1, interleukin (IL)‐1β, Gasdermin D, and induces pyroptosis, a form of cellular death in HT29 human colon adenocarcinoma cell line (Chen et al. 2022). In addition, luteolin induces apoptosis and blocks cell cycle progression by downregulating human telomerase reverse transcriptase protein (hTERT) in MDA‐MB‐231 breast cancer cell line. This suppression of hTERT modified the NF‐κB inhibitor α phosphorylation and subsequently affect the gene c‐Myc which is designated as master regulator of cell cycle (Huang, Jin, and Lan 2019). In the treatment of cancer cells, multiple mechanisms entails through which luteolin play a role to arrest cell cycle, induce apoptosis and leads to decrease in size and weight of tumor (Chen, Tien, et al. 2018). In MCF‐7 breast cancer cell line, luteolin also substantially enhanced the expression of miR‐34a and miR‐16 while significantly reduced the expression of miR‐21 that have resulted in decrease of cell viability, development of apoptosis in some cells during G0 or G1 phases of cell cycle by downregulating Bcl‐2 and upregulating BAX (Magura, Moodley, and Mackraj 2022). The NOTCH signaling hyperactivation that is predominantly involved in tumor development and progression during breast cancer was noted to be inhibited by luteolin via upregulation of miR‐34a, miR‐121a, miR‐139‐5p, miR‐246, miR‐224, and downregulation of miR‐155 (Sun et al. 2015). Luteolin (20 and 40 μM) also exerts inhibitory effects on triple‐negative breast cancer cells MDA‐MB‐231 through diverse mechanisms such as inhibition of matrix metalloproteinase‐9 expressions, suppression of migration and invasion, induced apoptosis via extrinsic and intrinsic pathways (Lee et al. 2019).

Luteolin also influences various pathways related to autophagy including nucleation and elongation that are necessary to prevent cancer progression. Luteolin affects the binding of endoplasmic reticulum (ER) chaperone which resultantly triggers stress sensors activation and stimulates autophagy (Ong et al. 2010). In addition, suppression of cancerous cells by luteolin is due to reduction in wingless‐related integration site (Wnt) signaling pathway that upregulated the fizzled class receptors. It was also reported that luteolin activates beclin 1 and caspases that increases the autophagy flux and microtubule‐associated protein (MAP) 1A/1B‐light chain 3 (LC3) puncta (Park et al. 2013; Lee, Park, Kang, et al. 2021). Another major role of luteolin in inhibition of cancer cell development is to initiate cell cycle arrest by decreasing phosphorylation of protein kinase B (Akt), which further dephosphorylates and triggers glycogen synthase kinase 3 (GSK‐3) and blocks the Wnt β‐catenin pathway. The cyclin D1 phosphorylation increases at Thr‐286 upon activation of GSK‐3 that enhances the destruction of proteasomes (Uekita et al. 2013). Luteolin has a prominent role in modulation of cleaving caspase and inhibition of cancer cells proliferation as it stimulates autophagy during sensitization of cancer cells. Furthermore, luteolin reduces the cancer cells proliferation upon MAPK protein activation that results in downregulation of P62 which resultantly initiates autophagy and stimulates FADD‐mediated apoptosis (Potočnjak et al. 2020). It also reduced tumor cells' survival and proliferation by regulating autophagy.

A recently identified calcium‐activated chloride channel protein, anoctamin 1 (ANO1), is proposed to have a fundamental role in proliferation of cells and tumorigenesis in most prevailing type of cancers that further linked with death in men. In prostate cancer cells, downregulation of ANO1 expression inhibits invasion, migration, and proliferation of cells. Luteolin with IC_50_ value of 9.8 μM significantly suppressed the ANO1 chloride channel activity and decreased the protein expression levels of ANO1 dose dependently (Seo et al. 2017). In prostate cancer, PC3 cells treated with luteolin showed poor cellular adhesion as well as increase in cell invasion through the AKT pathway via increasing E‐cadherin expression by inhibiting mouse double minute 2 (mdm2) gene (Zhou et al. 2009). Furthermore, administration of luteolin have resulted in reduction of cellular growth and triggered apoptosis in prostate cancer cells by upregulating death effector domain (DED)‐containing protein 2, a proapoptotic molecule, and downregulating miR 301 (Han et al. 2016). In prostate cancer cells PC3, suppression of mdm2 levels, induction of E‐cadherin, and prevention from invasion were noted to be associated with luteolin administration (Zhou et al. 2009). In another investigation by Han et al. (2018) explored that luteolin possess cancer preventive role against the proliferation of prostate cancer cell lines PC‐3 via suppressing the Wnt signaling pathways through upregulating the transcriptional FZD6 and inhibiting the stemness of these cells.

Different doses of luteolin (0.01, 0.1, 1, 10, 100 μmol/L) found to enhance calcified nodules content, promote cell viability, and ALP activity in periodontal ligament cells. In addition, runt‐related transcription factor 2, osteocalcin, bone morphogenetic protein 2, Osterix, and β‐catenin and cyclin D1 expressions were enhanced significantly after administration of lueolin at the dose of 0.01, 0.1, 1 μmol/L. Moreover, high‐luteolin administration lowered the suppressive effect on XAV939 inhibitor that suppressed the osteogenic differentiation‐related genes and calcification in human periodontal ligament cells (hPDLCs) (Quan et al. 2019).

The outcomes of various studies concluded that luteolin influences the factors related to metastasis and progression of tumor as it blocks the expression of matrix metalloproteinases (MMPs), proinflammatory cytokines (TNF‐α, NF‐κB, IL‐1, and IL‐6), and endothelial migration (Fang et al. 2018; Kang et al. 2017; Li, Chen, et al. 2017). Luteolin was also observed to possess an inhibitory role in release of chemokines by suppressing tumor‐associated macrophages and other associated cells related to immune system. For example, metastasis related to chemokine receptor C‐X‐C type 4 is actively inhibited by luteolin (Fang et al. 2018; Kang et al. 2017; Lin et al. 2008). Moreover, epithelial to mesenchymal transition exhibited a potent role in cancer metastasis which is efficiently suppressed by luteolin via targeting associated markers, signaling pathways, and several transcription factors (Hussain et al. 2021).

Inhibitory effects of luteolin related to invasion, migration, proliferation of cells is due to its activity to alter ERK, mammalian target of rapamycin (mTOR), PI3K/AKT, other related molecules functioning, and p38 signaling pathways (Lin et al. 2022). Luteolin reduces cellular invasion and metastasis by blocking EGFR‐signaling pathway. The luteolin ability to halt cell divisions and restricted focal adhesion kinase (FAK) activity are demonstrated to be important aspects in cancer treatment (Kim et al. 2013; Li, Lin, et al. 2017).

**FIGURE 3:**
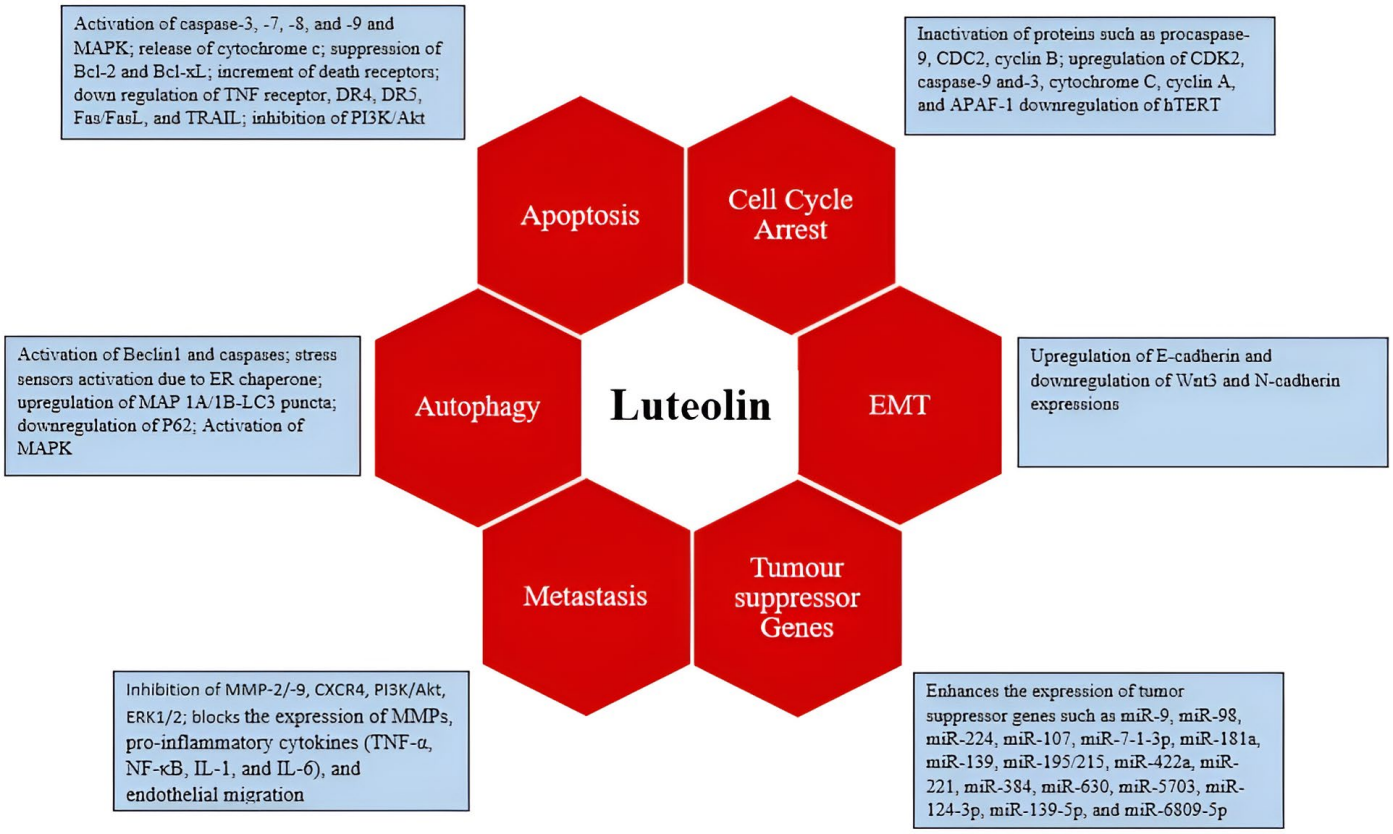
Role of luteolin in cell cycle arrest, apoptosis, autophagy, metastasis, EMT, and activation of tumor suppressor genes.

In studies involving several cancer cell lines have revealed that luteolin increased miR‐34 expression which is a crucial tumor suppressor gene (Jiang et al. 2018; Zhou et al. 2018). In addition, luteolin treatment enhances many other tumor suppressor genes expression including miR‐9, miR‐98, miR‐224, miR‐107, miR‐7‐1‐3p, miR‐181a, miR‐139, miR‐195/215, miR‐422a, miR‐221, miR‐384, miR‐630, miR‐5703, miR‐124‐3p, miR‐139‐5p, and miR‐6809‐5p, while decreasing the expression of some genes, including miR‐21, miR‐155 miR‐301, miR‐340, and miR‐224 (Figure 3; Mishan et al. 2021).

**FIGURE 4:**
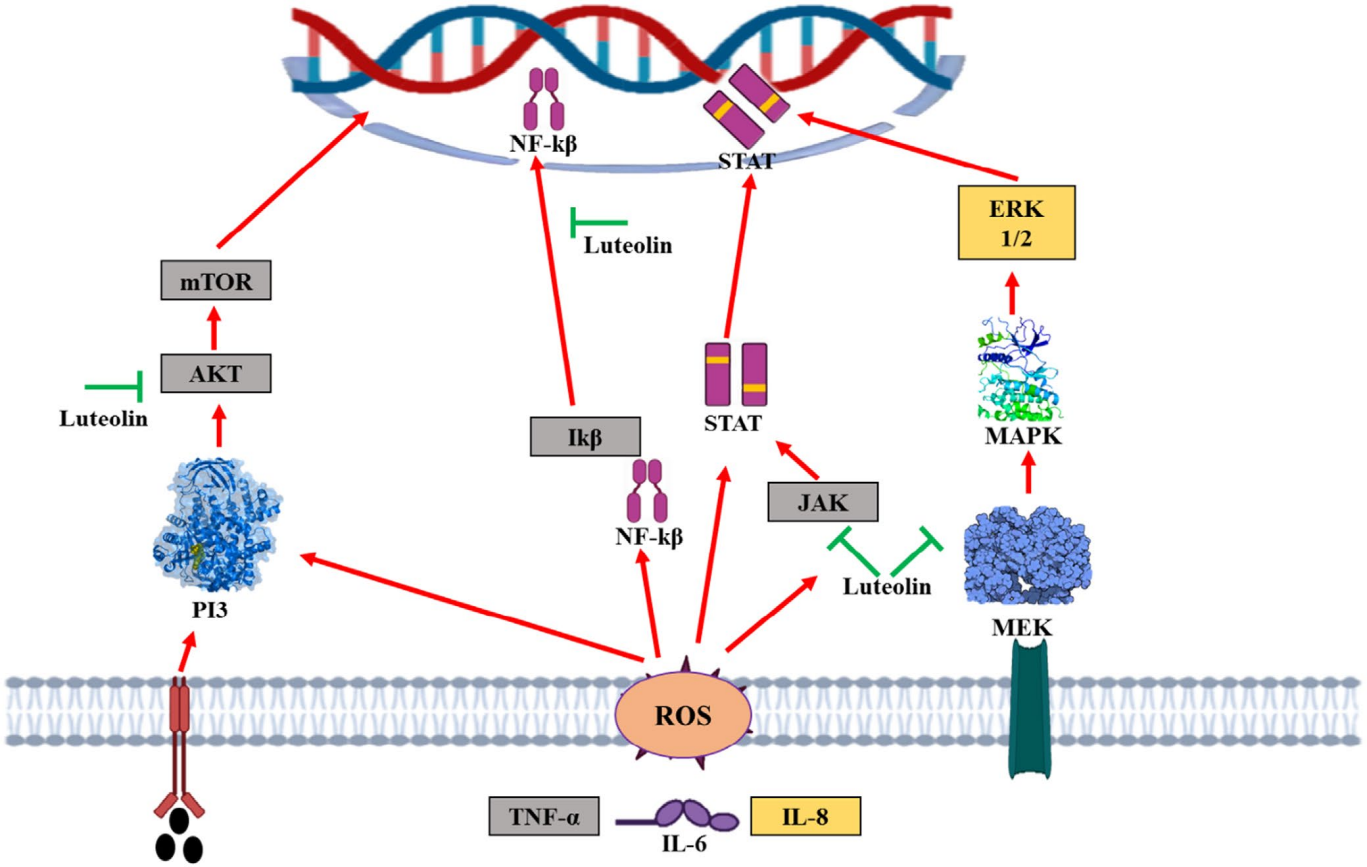
Various probable cancer‐causing inflammatory pathways modulated by luteolin.

**FIGURE 5:**
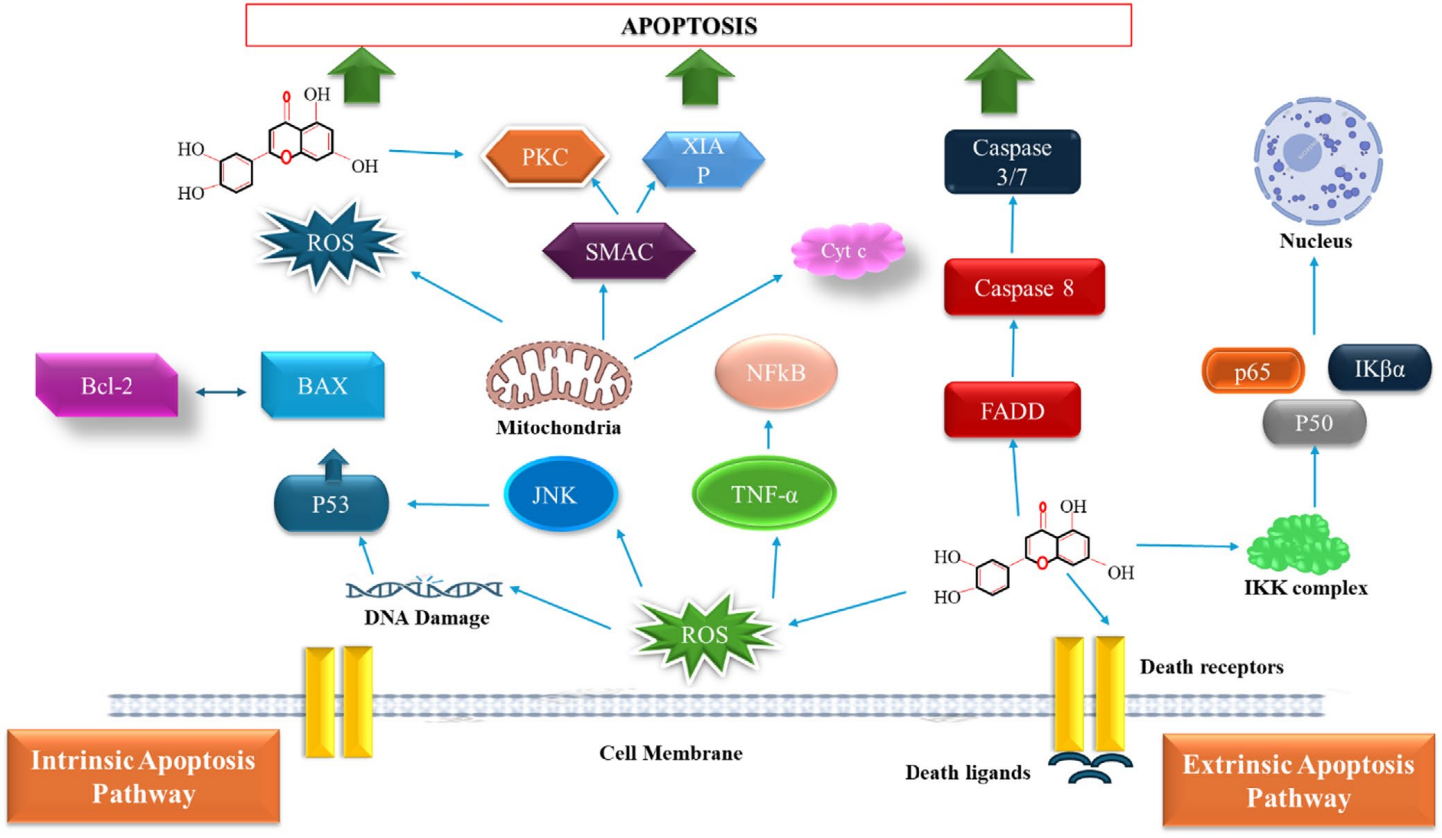
Signaling pathways modulated by luteolin.

Luteolin enhanced the miR‐34 gene expressions in gastric cancer cells, and its overexpression further boosted the cell susceptibility to luteolin (Zhou et al. 2018). The pancreatic ductal adenocarcinoma cells exposed to luteolin have resulted in reduction of cellular proliferation and upsurge in TRAIL induced antiproliferative effects by downregulating miR‐301‐3p (Moeng et al. 2020). In colorectal cancer cells, luteolin‐induced anticancer effects by modulating PTN via altering the miR‐384 expression. Luteolin have a role in inhibition of tumor growth in glioma cells by triggering autophagy and apoptosis through MAPK activation (Yao et al. 2019). In liver HL‐7702 cells, luteolin regulates Bcl‐2 level and enhances the BAX and caspase expressions (Ding et al. 2014). In glioblastoma cancer cells lines (U251 and U343 cells lines), reduction in cell viability, proliferation, migration, invasion, and colony formation of tumor organoids and patient‐derived glioma initiating cells (GICs) were reported after luteolin treatment (Yi et al. 2018). In another study by Zang et al. (2017), it reported that luteolin suppressed the invasion, migration, cellular proliferations, induction of apoptosis, reversion of epithelial‐mesenchymal transition (EMT), downregulation of N‐cadherin, Snail (zinc‐finger transcriptional repressor), mesenchymal biomarkers vimentin, and induction of epithelial biomarker E‐cadherin expressions in dose and time‐dependent fashion in experimental subjects. It also decreased the higher expression of Notch1, prevented from the formation of β‐catenin which further linked with activation of cancer cells stages. Luteolin have a crucial role in modulation of many inflammatory (Figure 4) and cell signaling pathways (Figure 5) related to cancer.

In many types of cancer cells including leukemia, pituitary tumor‐transforming gene 1 (PTTG1) is highly expressed and regulate cell proliferation. It was observed that PTTG1‐knockdown cells when exposed to luteolin results in sustained high levels of antiapoptotic proteins including p21, Bcl‐2, and Mcl‐1 and reduced the levels of apoptotic proteins. It was also found that in cancer cells with high expression of PTTG1 oncoprotein, luteolin was observed to play an essential role in reducing malignancy (Chen, Lai, et al. 2018). In another recent study reported by Wu et al. (2019) described that in GL261 cells, luteolin nano‐encapsulated modified poly‐(ethylene glycol)–poly‐(e‐caprolactone) markedly suppressed the tumor growth, and induced apoptosis in vitro and in vivo experimentation. Similarly, in colorectal cancer cells, luteolin downregulated the pleiotrophin expressions and up regulated the miR‐384 expressions in vitro and in vivo experiments (Yao et al. 2019). In nasopharyngeal carcinoma, repression of Zta and Rta genes, suppression of Epstein–Barr virus lytic protein expression, cell migration, and proliferation, along with Matrigel invasiveness, reduction in genomic instability, and tumorigenicity in experimental subjects were reported after luteolin treatment (Wu et al. 2016). According to Iida and coworkers, the anticancer potential of luteolin against human bladder cancer cell line T24 was due to induction cell‐cycle arrest at G2/M, downregulation of p‐S6, suppression of cell survival, upregulation of p21 and TRX1, reduction in ROS levels. They also observed reductions in tumor volume, enhancement in p21, and reduction in p‐S6 expression due to luteolin. In another study, N‐butyl‐N‐(4‐hydroxybutyl) nitrosamine was used to induce bladder cancer and it was noted that luteolin is markedly lowered the tumor dimension, inhibited cell proliferation and mTOR signaling, lowered the Ki67‐labeling index and p‐S6 expressions (Iida et al. 2020). In oral squamous cell carcinoma, luteolin administration influenced the cellular migration, invasion and lowered the p38 phosphorylation and MMP‐2 expression (Velmurugan et al. 2020). Luteolin exerted anticancer effects in HT29 cells as it inhibits nuclear factor‐erythroid‐2‐related factor 2 (Nrf2)/antioxidant response element (ARE) signaling pathway (Yang et al. 2020). Moreover, luteolin reduces Src/STAT3/S100A7 which inhibits the invasion and migration of squamous carcinoma (Fan et al. 2019).

Luteolin can be used to treat brain cancer due to ability of this molecule to easily cross the blood–brain barrier (Wruck et al. 2007). Rao et al. (2012) observed that luteolin suppresses the cell proliferation and induces apoptosis in different cancer cells through Akt, c‐Jun N‐terminal kinases, and p38. A potent tumor suppressor, miR‐106a‐5p, is newly discovered miRNA to have a role in suppression of epithelial‐mesenchymal transition is upregulated by luteolin and negatively regulated MMP2 and TWIST1 migration and have a critical role in anticancer strategies (Wang et al. 2024).

Several in vitro and in vivo cancer cells trials exhibited that luteolin reversed the multidrug resistance. In ovarian cancer cells, luteolin chemosensitizes the cells through repressing the epithelial‐mesenchymal transition markers and traits, epithelial‐mesenchymal transition, and integrin b1 (Dia and Pangloli 2016). The in vitro anticancer effect of luteolin in dosage dependent manner in cisplatin‐resistant CAOV3/DDP ovarian cancer cell lines of mice xenotransplant model was determined by Wang and colleagues. They found that luteolin suppressed the proliferation rate, enhanced the cisplatin‐induced downregulation of Bcl‐2 expression, suppressed the migration and invasion stages whereas in vivo study, luteolin also lowered tumor growth and induced apoptotic cellular deaths (Wang et al. 2018).

In recent research on ovarian cancer stem cells, luteolin was found to bind directly with KDM4C that results in decrease stemness of these cells by transcriptional inhibition of PPP2CA/YAP axis (Li, Li, et al. 2023). Luteolin treatment could increase cytokine production, cell chemotaxis, and T‐cell activation. Moreover, high ratio of CD8+ T lymphocytes was maintained by luteolin in the tumor tissues, peripheral blood, and spleen (Cai et al. 2024). In addition, luteolin was found to inhibit tumor resistance (hinder migration invasion, inhibit cellular proliferation, induce apoptosis and cell cycle arrest, and spherification of stem cells and EMT) (Yang et al. 2024). Luteolin have the ability to bind with the active sites of AKT, Src, and focal adhesion kinase by inhibiting PI3K/AKT signaling pathways and in this way enhanced drug chemosensitivity (Yang et al. 2024). Luteolin was also observed to modulate gut microbiota which reduce the number of tumors in case of colorectal cancer by enhancing the number of health‐related microbiota and reduced the microbiota related to inflammation (Pérez‐Valero et al. 2024). The nanoparticles encapsulation of luteolin was found to be more effective in inhibition of PC3 and HeLa cells from proliferation and migration in experiments related to prostate cancers and anticervical tumors (Ding et al. 2024). Luteolin has wide spectrum therapeutic benefits to control and target cancer cells and could be utilized as an effective anticancer agent.

### 3.2 Cardioprotective Role of Luteolin

**FIGURE 6:**
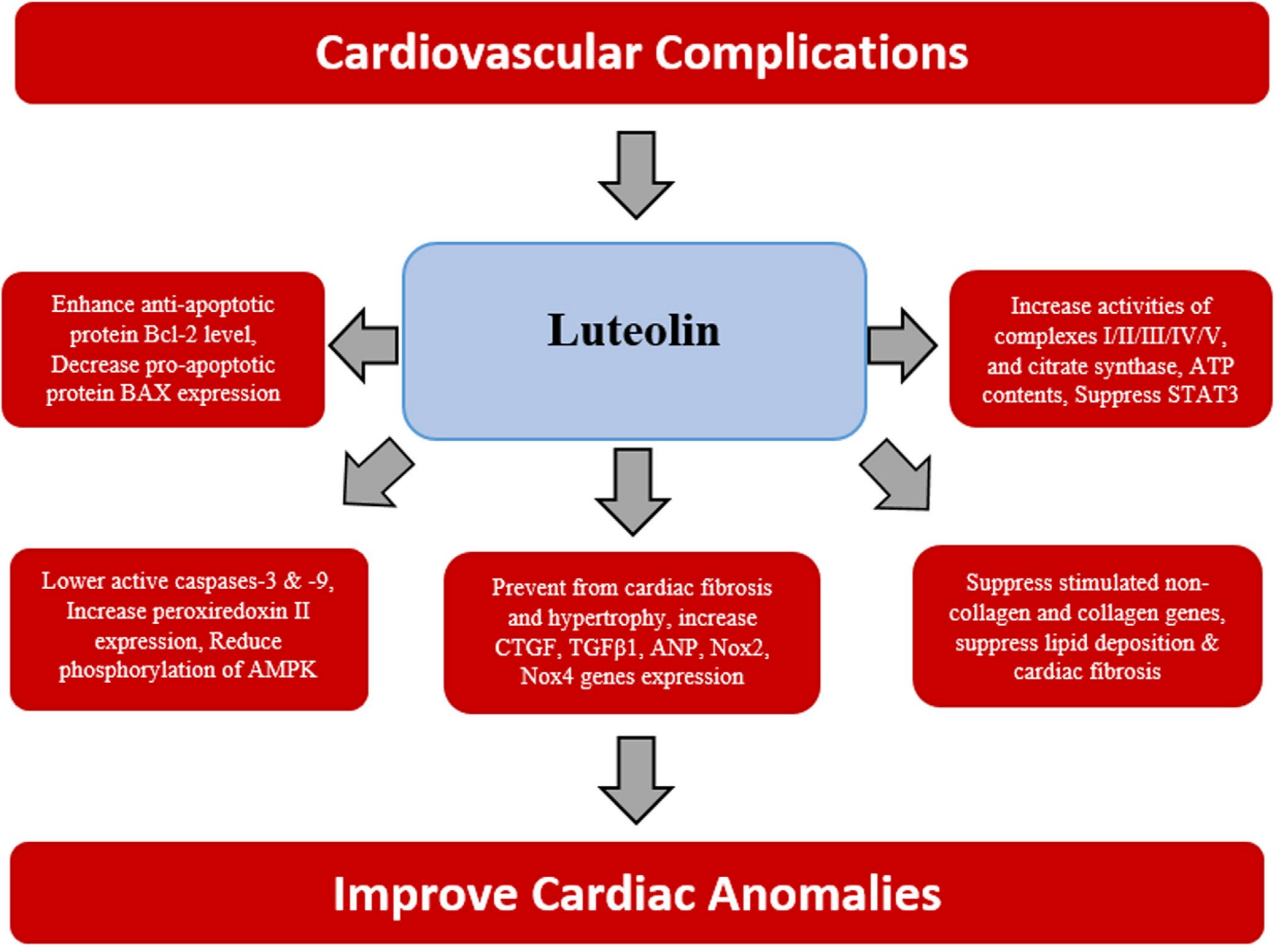
Target molecules of luteolin to improve cardiovascular complications.

The cardioprotective potential of luteolin is primarily due to its antioxidative, anti‐inflammatory, and antiapoptotic properties (Pan et al. 2022). In vivo and in vitro trials highlighted that luteolin exerted substantial effects on cardiovascular complications through various pathways (Figure 6) such as enhancing antiapoptotic protein Bcl‐2 level, improving cardiac function, lowering active caspases‐3 and ‐9, decreasing the proapoptotic protein BAX, and increasing peroxiredoxin II expressions (Wei et al. 2018).

High mortality rate is linked with myocardial infarction (MI) with characteristic left ventricle dysfunction and chamber dilation. The significant improvement in cardiac function was observed after luteolin administration by modulating cardiac enzyme activity and inhibition in release of inflammatory cytokines after MI. Furthermore, in the neonatal cardiomyocytes, autophagic flux was improved as indicated by less accumulation of aggresomes, P62, and more autophagosomes puncta after hypoxia in groups pretreated with luteolin. Luteolin upregulated autophagy in the cardiomyocytes involved in simulation of MI injury. In addition, luteolin increased complexes I/II/III/IV/V activities, citrate synthase activity, adenosine triphosphate content, and mitochondrial membrane potential in these cardiomyocytes (Hu et al. 2016).

In a study, it was reported that diet based luteolin (0.035%) in Ang II infected experimental rats prevented from cardiac fibrosis and hypertrophy along with enhancement of level of gene expressions such as CTGF, TGFβ1, ANP, Nox2, and Nox4. Moreover, oral pretreatment with high‐luteolin diet restored luteolin as well as plasma B‐type natriuretic peptide (BNP) induced by Ang II. Luteolin pretreatment suppressed the phosphorylation of JNK and H_2_O_2_‐induced TGFβ1 expression in cultured rat cardiac fibroblasts (Nakayama et al. 2015). Suppression of stimulated noncollagen extracellular matrix genes (elastin, fibrillin‐1, collagen triple helix repeat containing 1, and connective tissue growth factor) and collagen genes (Col1a1, Col1a2, Col12a1, and Col3a1), prevention from myocardial injury and fibrosis, blocking of isoproterenol (ISO)‐stimulated expression of the genes (Rac2, Ncf1, Ncf4, Cyba, and Cybb), and reversion of ISO‐altered expression of microRNAs, that is, miR‐21, miR‐29c‐3p, miR‐29c‐5p, miR‐30c‐3p, and miR‐30c‐5p, were reported after intraperitoneal (i.p) administration of luteolin (5–40 mg/kg/d) in mice with ISO‐induced fibrosis and myocardial injury (Ning et al. 2017).

Luteolin in septic heart tissues as cardioprotective agent has been found to prevent myocardial injury and exhibited numerous functions such as improvement in cardiac functions, inhibition of cardiac apoptosis, enhancement in autophagy, alleviation of mitochondrial injury, reduction in oxidative stress, and attenuation of the inflammatory responses, as well as reduction in phosphorylation of AMP‐activated protein kinase (AMPK) (Wu et al. 2020). Similarly, amelioration of cardiac activity, stability and expression of sarcoplasmic reticulum Ca^2+^‐ATPase 2a (SRCA2a), sumoylation of SRCA2a, specificity protein 1 (Sp1), enhancement of phosphorylation of Akt, phospholamban (PLB), and improvement in contractility and Ca^2+^ transients, transcriptions of SUMO1 and SRCA2a, amelioration of myocardium fibrosis, suppression of posphatidylinositol 3 kinase/Akt (PI3K/Akt) pathway, and upregulation of caspase‐3/cleaved caspase‐3, Bcl‐2/BAX expression ratios were reported after luteolin treatment in vitro and in vivo trials. In addition, luteolin has been found effective to prevent heart failure (Hu et al. 2017). It also prevents hypertrophy and autophagy, lowers cell viability, α‐actinin, and LC3 expression levels. Luteolin enhanced the cell viability of lipopolysaccharide (LPS)‐treated cardiomyocytes and prevented cardiomyocyte hypertrophy and autophagy induced by LPS in rat model. In these cardiomyocytes, luteolin also decreased expression levels of LC3 and α‐actinin. In addition, the expression levels of atrial and brain natriuretic peptide which are cardiac hypertrophy associated markers were effectively controlled by luteolin. The suppression of mRNA and protein expression levels of Wnt signaling pathway‐associated genes and LPS‐mediated autophagy were also observed to be associated with luteolin administration. Moreover, the formation of autophagosomes and inhibition of cardiomyocyte hypertrophy induced by LPS was due to luteolin impact on silencing of β‐catenin (Li et al. 2019).

Recently, luteolin (50 mg/kg and 100 mg/kg) was given to experimental rats to notice preventive effects of this molecule against doxorubicin (DOX)‐induced cardiotoxicity. Luteolin significantly ameliorated heart weight changes, DOX‐induced weight loss and DOX‐induced cardiotoxicity dose dependently. The changes in oxidative stress and biochemical parameters were also improved due to luteolin. Luteolin preventive role was mainly due to regulation of expression levels of mRNA and proteins related to apoptotic markers caspase‐3, Bcl‐2, and BAX. The activation of Akt/Bcl‐2 signaling pathway was noted in network analysis,which showed that it was activated. Specifically, they were involved in the AKT/Bcl‐2 signal pathway. DOX‐induced cardiotoxicity was attenuated by luteolin via inhibiting the activity of PH domain leucine‐rich repeats protein phosphatase 1 (phlpp1) that imparts positive impact on the Akt/Bcl‐2 pathway regulations (Zhang et al. 2020).

The myocardial I/R injury is mainly due to Ca^2+^ overload in SR by excessive uptake of Ca^2+^ from the cytosol which is mediated by SRCA2a. The reduction in expression and activity SRCA2a is primarily important to heighten Ca^2+^ overload (Akolkar et al. 2012; Talukder et al. 2008). In a vitro trial of I/R injury, luteolin has been found to lower the lactate dehydrogenase release, myocardial infarct size, and apoptosis. Along with, decrease in SRCA2a after myocardial I/R injury was suppressed by luteolin in vivo experimentation (Nai et al. 2015).

Moreover, luteolin also considerably caused increase in apoptosis regulator Bcl‐2 and reduction in expression levels of apoptosis regulator BAX as well as lowering the concentration of urea, creatinine, malanodialdehyde (MDA), and lactate dehydrogenase upon luteolin treatment. Under hypoxia conditions, it also exhibited suppression of ventricular myocytes L‐type calcium currents dose dependently (Yan et al. 2019). DOX has been known as an effective antineoplastic agent via triggering cardiac anomalies including myocardial injury, tachycardia, and heart failure (Wenningmann et al. 2019). Xu and coworkers explored the cardioprotective role of luteolin in adult mouse cardiomyocytes through administrating the dose of 10 μM in DOX‐induced cardiomyocyte. They observed induction of apoptosis, reactive oxygen development, promotion of mitochondrial autophagy, loss of mitochondrial membrane potential, facilitation in phosphorylation of dynamin‐related protein 1 (Drp1) at Serine 616, and upregulation of the transcription factor EB (TFEB) expression after luteolin administration (Xu et al. 2020).

In a study on C57BL/6 mice with induced diabetic cardiomyopathy, luteolin minimized the production of ROS, enhanced the Nrf2 and heme oxygenase‐1 (HO‐1) levels; reduced the oxidative damages and inflammation which is crucial to maintain heart functioning under these conditions (Li et al. 2019). Luteolin reduced cardiomyocyte apoptosis, attenuated myocardial injury, enhanced SERCA2a transcriptional activity, and improved left ventricular function by upregulating Sp1 expression which is a key antiapoptotic mechanism that increases the cardiomyocytes contractile capacity (Hu et al. 2020). In ApoE−/− mice fed with high‐fat diet, luteolin substantially reduced atherosclerosis by reducing inflammation. In this trial, oxidized low‐density lipoproteins including the mRNA production of pro‐inflammatory mediators such as IL‐6, ICAM‐1, VCAM‐1, and TNF‐α were used to induce inflammation, which was reduced by luteolin via suppressing STAT3, a critically important therapeutic target in controlling atherosclerosis. Hence, luteolin might be a potential candidate in novel drugs development strategies against atherosclerosis (Ding et al. 2019).

Reduction in hyperlipidemia‐induced cardiac damage were observed after luteolin treatment via suppression of lipid deposition (LOX‐1 and CD36) and cardiac fibrosis (MMP2, MMP9, Collagen I, Collagen III, and TGF‐β) (Dong et al. 2023). The cardioprotective role of luteolin is well corroborated in several cell lines and animal models; hence, it might be a potential molecule to be helpful in treatment of cardiovascular complications and improving heart functioning in humans. Its regular dietary intake could play a role in the prevention of life‐threatening heart diseases.

### 3.3 Antidiabetic Effects of Luteolin

**FIGURE 7:**
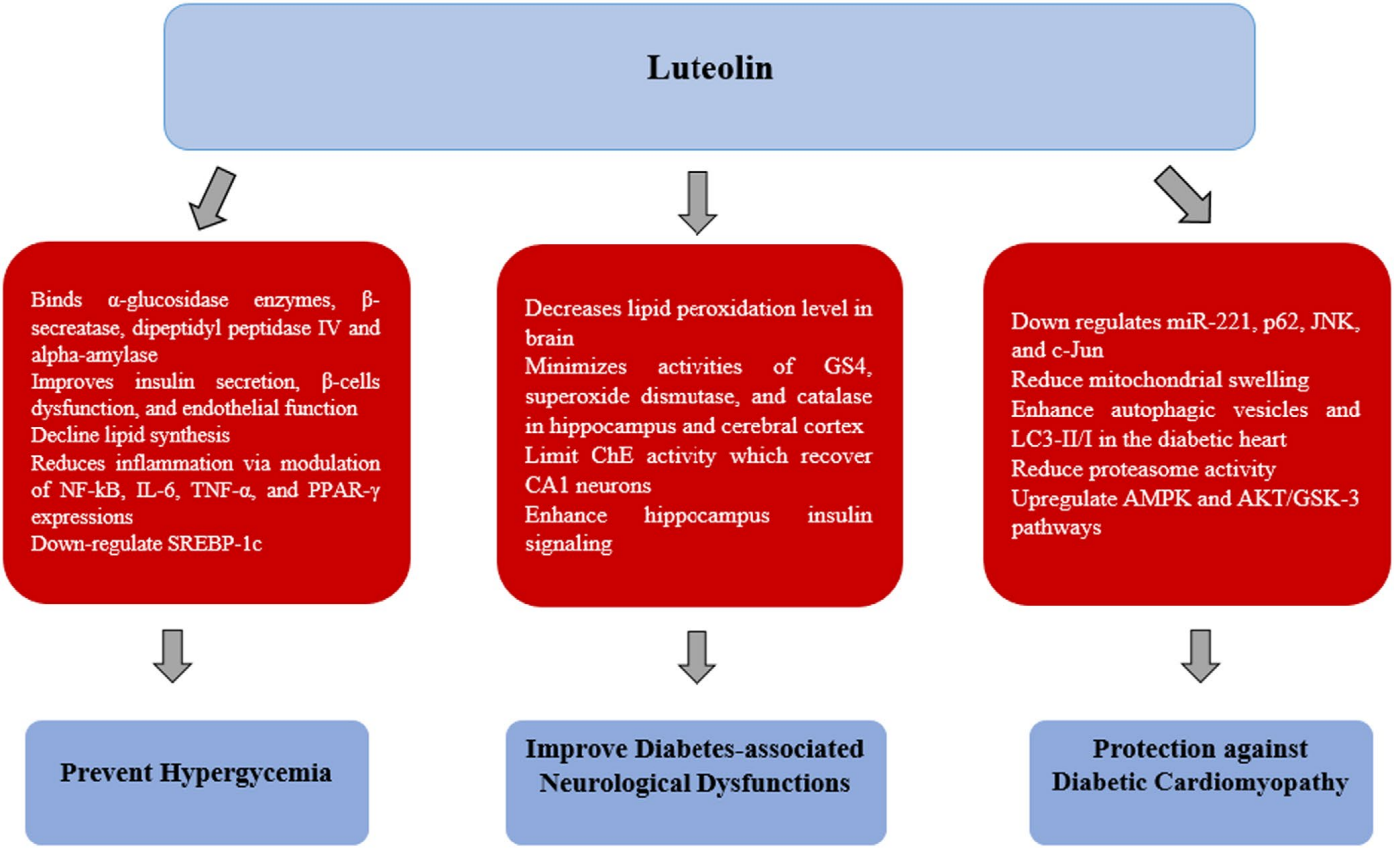
Molecular mechanism of luteolin to manage diabetes and related complications.

Diabetes is a major disease that globally affects the health of populations and caused severe life‐threatening illnesses (Lin et al. 2020). Multiple studies established a link between luteolin consumption and avoidance from diabetes (Figure 7). Luteolin can be considered as potent antidiabetic agent (Tuorkey 2016). Luteolin exhibits highly effectiveness, noncompetitiveness and acts as potent reversible inhibitor of α‐glucosidase (Djeujo et al. 2022), as it has strong affinity to bind with α‐glucosidase enzymes and β‐secretase enzyme (BACE1) whether present in high or low concentration (Wagle et al. 2019). In a molecular docking study, it was revealed that luteolin binds dipeptidyl peptidase IV and alpha‐amylase efficiently. Thus, it provides prevention from glucose optimization and then binds to Forkhead box protein O1 and glutamine‐fructose‐6‐phosphate aminotransferase which highlighted that it has a potential role in avoiding hyperglycemia, hence, luteolin proved to be a potential molecule in controlling Type 2 diabetes (Davella and Mamidala 2021). Luteolin and its derivatives (O‐ and C‐glycosides) possess several antidiabetic effects including improvement in blood glucose, insulin, HOMA‐IR, and HbA1c levels, and decline in lipid synthesis (Zang, Igarashi, and Li 2016). Likewise, luteolin ameliorated diabetes by enhancing insulin secretion, improving β‐cell dysfunction, insulin resistance, endothelial function, and reducing inflammation via modulation of NF‐kB, IL‐6, TNF‐α, and PPAR‐γ expressions, inhibition of α‐glucosidase and dipeptidyl peptidase‐4 activities and downregulation of SREBP‐1c (Shehnaz et al. 2023; Chang and Yue 2024).

The diabetes that continue for long‐term may impact the neurons present in the cerebral cortex and luteolin administration reverse this negative impact due to its ability to decrease lipid peroxidation level significantly which was observed to be accumulated excessively in rat brain during diabetic condition, and also minimizes the activities of Growth Stage 4 (GS4), superoxide dismutase, and catalase in the hippocampus of rats and cerebral cortex after luteolin administration. Luteolin limited ChE activity and helps to recover CA1 neurons by reducing neural death via luteolin antioxidant activities. On the other hand, increase in ChE activity causes neurological dysfunction and progressive cognitive impairment during diabetes. In this way, luteolin has a pivotal role in improving diabetes related adverse effects (Liu et al. 2013). The insulin resistance in brain may also reduce due to luteolin. In a study, it was observed that hippocampus insulin signaling was potentiated as well as glucose metabolism was enhanced along with improvement in β‐cell functioning and increase in hepatic insulin sensitivity after treatment with luteolin (Park et al. 2018).

During prolonged duration of diabetic complication, heart muscles may also be affected and caused myocardial I/R or other damage mainly because of increase in oxidative stress. It was revealed that heart muscle damages and level of oxidative stress significantly reduced after treatment with luteolin as this phytochemical redirect the oxidation reactions via triggering Nrf2‐dependent sestrin2 regulator to generate positive feedback loop (Zhou et al. 2021). Luteolin also provide protection against diabetic cardiomyopathy by down regulation of miR‐221, p62, JNK, and c‐Jun, reduction of mitochondrial swelling, enhancing autophagic vesicles and LC3‐II/I in the diabetic heart (Xiao et al. 2023). In another experiment, luteolin was found to protect against diabetic cardiomyopathy by reducing proteasome activity and upregulation of AMPK and AKT/GSK‐3 pathways (Zhang et al. 2023).

Luteolin actively inducing antioxidants in diabetic nephropathy and imparts renoprotective effects by enhancing the HO‐1 expression. Luteolin also provide prevention from morphological damages to the kidney incurred during diabetes mellitus (Wang, Wuniqiemu, et al. 2021). Furthermore, in primary mouse adipose cells, luteolin was reported to have a role in initiation of insulin action and boosting peroxisome proliferator activated receptor γ (PPAR‐γ) target genes expression (Ding, Jin, and Chen 2010). Luteolin decreases microautologous fat transplantation (Maft), an insulin gene trans‐activator in dysfunction pancreatic β cells, which enhances insulin secretion in uric acid through NF‐κB (Ding et al. 2014). In adipose tissues, M1‐like macrophage polarization was altered by luteolin that helps to improve adipose tissue inflammation and insulin resistance (Baek et al. 2019).

### 3.4 Luteolin Against Obesity

In the management of obesity, luteolin as dietary supplement has proved to be a desirable molecule. In a study, it was found that luteolin increased the metabolomic rates and helped in reduction of diet‐induced obesity by triggering the AMPK/peroxisome proliferator activated receptor gamma coactivator 1‐α (PGC1‐α) pathway (Zhang et al. 2016). Likewise, in a study on diet‐induced obesity mice, it was reported that cholesterol level was reduced due to luteolin by regulating cholesterol efflux regulators such as scavenger receptor class B member 1 (SR‐B1), ATP‐binding cassette (ABC) transporter G1, and liver X receptor α (LXRα) (Park et al. 2020). In another study, C57BL/6N mice were used to observe impact of luteolin from artichoke leaves on obesity parameters. The results indicated that luteolin has a profound role in reduction of adverse effects incurred due to obesity even in high‐fat fed mice (Kwon et al. 2018a). Moreover, luteolin supplementation reduced macrophage infiltration and dysregulation of adipokine/cytokine due to capabilities of this molecule to change Toll‐like receptor (TLR) signaling pathways (Kwon et al. 2018b). Furthermore, luteolin and its derivatives are valuable moieties against obesity due to their regulatory activities against differentiation of adipocytes by modulating the tissue factor PPAR‐γ (Park et al. 2009). The obesity adipocyte inflammation is reduced by administration of luteolin which blocks the activity of proinflammatory mediators in macrophages such as monocyte chemoattractant protein, TNF‐α, and NO (Ando et al. 2009). Hence, various cellular mechanisms are involved through which luteolin helps to control and manage obesity in animal models, which could be translated to treat human obese subjects. Recently, nutraceutical products containing luteolin was found to be helpful in managing body weight, lipid and glycemic parameters, and physiological aspects related to hepato‐ and cardiometabolic functions in human obese and preobese subjects (Terzo et al. 2023). Furthermore, luteolin enhances adipose tissue thermogenesis, increases systemic energy expenditure, inhibit lipogenesis and inflammation in adipose tissues and improves obesity and related chronic ailments (Zhang et al. 2024).

### 3.5 Anti‐Inflammatory Potential of Luteolin

Luteolin is a well‐known anti‐inflammatory agent (Tuorkey 2016). Chen and coworkers explored anti‐inflammatory role of luteolin in lipopolysaccharides induced mice. They observed that luteolin has been found to prevent from the disease severity of chronic pharyngitis, granuloma, and ear edema by inhibiting the proinflammatory cytokines (TNF‐α, IL‐6, and IL‐12) activity in macrophages (Chen, Tien, et al. 2018). They also observed that luteolin supports polarization of macrophage M2 by suppressing the NF‐κB, enhancing mannose receptor C Type 1 (Mrc1) and arginase (Arg1) expressions and activating interferon regulatory factor (IRF) 1, and IRF5 expressions. Inflammatory chronic pharyngitis was relieved by luteolin via polarization of M1 macrophage and suppression of NF‐κB pathway (Chen, Tien, et al. 2018).

Luteolin was found to be effective in ocular inflammation as it significantly reduced protein levels, number of inflammatory cells, clinical severity scores, and levels of prostaglandin (PG) E2, TNF‐α, and nitric oxide (NO) in the AqH and ocular tissue after i.p injection of 10 mg/kg luteolin (Kanai et al. 2016). Inflammatory cytokines, for instance, TNF‐α and IL‐1β are responsible to promote inflammation via increasing their secretions in human mast cells (MCs)‐1. Luteolin significantly impedes in the production of inflammatory cytokines, reduced the release of histamine from rat peritoneal MCs, and suppressed the vascular permeability and scratching behavior in experimental subjects' dose dependently (Jeon et al. 2014).

Luteolin was found to be effective in providing protection against acute lung injury induced by LPS as luteolin caused attenuation in leukocyte infiltration and protein extravasation, formylmethionyl‐leucyl‐phenylalalnine (fMLP)‐incited neutrophil chemotaxis (IC_50_ 0.2 ± 0.1 μmol/L) and respiratory burst (IC_50_ 2.2 ± 0.8 μmol/L) (Lee et al. 2010). Moreover, blockade of phosphorylation of Akt, ERK, and MAPK/ERK kinase 1/2 (MEK) in LPS‐ and fMLP‐stimulated neutrophils were reported after luteolin treatment (Lee et al. 2010). In another investigation, Dirscherl et al. (2010) strengthened the previous findings and found that luteolin triggered changes in LPS‐activated microglia and inhibited the proinflammatory marker expressions, blockade the proapoptotic and proinflammatory genes expression. Conversely, coincubated with luteolin, notable decrease in NO secretion and neurotoxicity was noted on 661 W photoreceptor cultures in LPS‐activated microglia (Dirscherl et al. 2010). A peer group of researchers investigated the anti‐inflammatory role of luteolin against pam3CSK‐ and LPS‐treated macrophage cell line (RAW264.7) and found that multiple processes are involved such as blockade of Src kinase and Syk kinase, inhibition of PGE_2_, NO and, nuclear translocation of NF‐*κ*B (p50 and p65), suppression of TNF‐*α*, and cyclooxygenase‐2 (COX‐2) levels, levels of inducible NO synthase (iNOS) mRNA transcripts without affecting MAPK to cease inflammation (Lee et al. 2015). In chronic obstructive pulmonary disease, luteolin alleviates oxidative stress and inflammation via inhibition of NOX4‐mediated NF‐κB signaling pathway and levels of inflammation factors (Li, Wang, et al. 2023).

Luteolin therapy inhibits TNF‐α and IL‐6 production, promotes expression of RNA binding protein tristetraproline (TTP), and suppresses the phosphorylation of p38 and MAPK‐activated protein kinase‐2 (MK‐2) (Wu et al. 2013). Moreover, luteolin supplementation at the rate of (0.5 μM) markedly prevented from the vascular inflammation in a permanent human endothelial cell line EA.hy926 via various mechanisms such as suppression of TNF‐α‐mediated monocytes adhesion, TNF‐α‐induced monocyte chemotactic protein‐1 (MCP‐1), ICAM‐1 and VCAM‐1 expressions and inhibition of TNF‐α‐induced IκBα degradation, transcriptional activity NF‐κB, subsequent nuclear translocation of NF‐κB p65, and expression of IκB kinase ß (IKKß) whereas in animal experimental study of C57BL/6 mice which supplemented by 0.6% luteolin for 3 weeks and caused suppression of TNF‐α‐mediated increase in circulating levels of soluble ICAM‐1, CXCL1/KC, and MCP‐1/JE. Luteolin provide protection to endothelial lining of aortic intima layer from eruption by reducing TNF‐α‐mediated monocytes adhesion and preserved monocyte‐derived F4/80+ macrophages and elastin fibers in aorta of mice treated with TNF‐α (Jia et al. 2015).

Sepsis, a syndrome of systemic inflammation, has resulted in vital organ damage. In different organs, the onset of sepsis is mediated by different pathways. A NALB/c inbred male mice was studied as in vivo model of sepsis and observed that luteolin was found to have a role in inhibition of phagocytosis of macrophages, downregulation in expression of myeloid differentiation factor 88 (MyD88) and TLR4. It was also observed that luteolin regulated the expression of cytokines including TNF‐α, IL‐1β, IL‐6, and IL‐10 and reduced the level of PPAR‐γ, activator of transcription protein (STAT) and signal transducer. Therefore, sepsis inflammation could be effectively counteracted by luteolin due to its role in inhibition of PPAR‐γ/STAT/MyD88 pathway (Miao, Li, and Li 2018).

### 3.6 Hepatoprotective Role of Luteolin

Lipopolysaccharides exhibits hepatic injury via increasing the concentrations of serum ALT, AST, TNF‐α, cyclooxygenase levels and its transcription factors, activator protein (AP)‐1, and NF‐κB, decreasing the levels of phase II enzymes and activating NF‐E2 p45‐related factor (NRF)‐2 while luteolin administration to experimental subjects revert these changes (Park and Song 2019). It was explored that oral gavage administration of luteolin (100 mg/kg/day) for three consecutive days provided protection against hepatic injury induced by methamphetamine through modulating the cytochrome P450, oxidative phosphorylation, and certain signaling pathways (Qu et al. 2020). Furthermore, intraperitoneal lead acetate treatment (20 mg/kg, daily) has been used to induce liver toxicity in experimental subjects via enhancing the hepatic residues, serum liver enzymes (AST, ALT, and total bilirubin), decreased the concentrations of antioxidant enzymes, lowered antioxidant proteins such as Nrf2 and heme oxygenase‐1, enhanced the NO and MDA level, as well as showed improvement in iNOS, TNF‐α, IL‐1β, and NF‐*κ*B, downregulated the Bcl‐2, and increased BAX and caspase‐3 proteins. On other hand, luteolin oral administration at the rate of (50 mg/kg, oral, daily) reversed these changes (Al‐Megrin et al. 2019). The liver lesions improve significantly through various mechanisms such as luteolin minimizes the unnecessary accumulation of extracellular matrix, reduces oxidative stress, inhibits inflammatory markers, and effectively regulates lipid balance (Yao et al. 2023).

In Kunming mice, the administrated mercuric chloride at the rate of 4 mg/kg caused anemia, induced hepatotoxicity, enhanced the levels of free radicals, regulated the hepatocyte viability, increased the inflammatory proteins such as TNF‐α, NF‐κB, mTOR, Sirt1, p53, and upregulated the p38 MAPK activation as well as lowered the antioxidant defense system based on HO‐1, KLF9, Nrf2, kelch‐like enoyl‐coenzyme A hydratase (ECH)‐associated protein 1 (Keap1), NADPH quinine oxidoreductase 1 (NQO1), and while supplementation of luteolin (100 mg/kg) 24 h in vitro reversed these changes (Yang et al. 2016). The luteolin provide protection against oxidative stress induced by mercury metal in liver by enhancing Nrf2 levels, upregulating nicotinamide adenine dinucleotide phosphate (NADPH), quinone oxidoreductase 1, heme oxygenase‐1, and its downstream factors, reducing expression of NF‐κB and p53, decreasing expression of Bcl‐2 and Bcl‐xL, as well as enhancing BAX level which further enhanced the BAX/Bcl‐2 ratio (Zhang, Xing, et al. 2017; Castellino et al. 2019).

Antioxidative role of luteolin with 1 μM doses was investigated against HepG2 and WRL hepatic cell lines by Wu and their fellows and reported prevention from nuclear translocation of sterol regulatory element binding protein (SREBP)‐2, suppression in the level of SREBP‐2, enhancement in AMPK activation, decrease in mRNA and protein expression of SREBP‐2 at transcriptional level as well as reduction in transcription of HMG CoA reductase (HMGCR), following luteolin treatment (Wong, Lin, and Leung 2015).

### 3.7 Renal Protection Associated With Luteolin

The most prevalent cause of acute kidney injury is renal I/R injury which is linked to high mortality rate. Being a potent antioxidant agent, luteolin intragastric dosage of 40 mg/kg for 3 days prevented the kidney injury in Sprague–Dawley rats through various mechanisms such as decreasing the concentrations of MDA level, myeloperoxidase (MPO) levels, 8‐oxo‐deoxyguanosine, urea nitrogen and creatinine level, along with enhancement in antioxidant enzymes. In I/R rats, luteolin attenuated the increased expression levels of IL‐1β, IL‐6, TNF‐*α*, NF‐*κ*B, and renal high mobility group box‐1 (HMGB1). Moreover, significant reduction in renal cell apoptosis and ER stress induced by renal I/R injury was observed after luteolin treatment (Hong et al. 2017). Whereas these renal damages were considerably lowered in luteolin pretreatment groups with significant improvements in renal functions, LPS‐induced renal injury has resulted in production of excessive serum creatinine, blood urea nitrogen, increased tubular necrosis and oxidative stress, as well as rapid renal damage in mice. The expressions of ICAM‐1, MCP‐1, cleaved caspase‐3, TNF‐α, IL‐1β, and NF‐κB were increased after induction of LPS while luteolin pretreatment reversed these disturbed expressions (Xin et al. 2016).

In male Swiss albino mice, luteolin prevented from renal I/R injury through substantial decrease in the levels of IL‐6, IL‐1β, and TNF‐α in comparison with I/R groups without luteolin. It was also revealed that luteolin reduces the number of apoptotic cells and enhances the cell viability of damaged renal tissues. The Bcl‐2 expression was increased while BAX and caspase‐3 expressions decreased after luteolin pretreatment (Liu et al. 2017). In Wister rats, pretreatment with 50 mg/kg oral administration of luteolin was observed to protect from renal damage through various detoxification mechanisms including antiapoptotic, anti‐inflammatory, and antioxidant activity (Albarakati et al. 2020). In another experiment, carbon tetra chloride induced hepatoxicity was reduced by luteolin via boosting antioxidant enzymes activity (Yan et al. 2019). Furthermore, renal injury was observed to be attenuated in hyperuricemic nephropathic mice by administering luteolin through inhibition of XO activity, modulation of uric acid transporters, as well as activation of Nrf2 HO‐1/NQO1 antioxidant pathways and renal SIRT1/6 cascade (Yu et al. 2024). Perfunctorily, KIM‐1, caspase‐3, and renal ATP‐binding cassette subfamily G isoform 2 protein expressions were reduced due to luteolin (Yu et al. 2024).

### 3.8 Luteolin Protects Brain

Luteolin protects traumatic brain injury in mice by preventing inflammatory responses. In addition, neuroprotective effects of luteolin are mainly due to induction of autophagy (Xu et al. 2014). The secondary brain injuries such as neuronal apoptosis, imbalance in brain water contents, and neurological deficits are suggestively ameliorated by luteolin (Xu et al. 2014). The dopaminergic neurons are also protected by luteolin via activation of microglia, decreasing neuroinflammation and oxidative damage, as well as improving neurotrophic potential (Patil et al. 2014). The protection from ischemic damage to brain due to luteolin might be related to upregulation in claudin‐5 expression along with reduction in apoptosis and oxidative stress (Qiao, Zhang, et al. 2012). Luteolin plays a role in reducing damages to the brain incurred due to occlusion of middle cerebral artery (MCA) by upregulation of ERK expression and downregulation of TLR4, TLR5, p38MAPK, and NF‐κB (Qiao, Dong, et al. 2012). Luteolin prevented from the secondary brain injury induced by intracerebral hemorrhage developed through upregulating the neural oxidative stress through multiple pathways such as alleviation of brain edema, amelioration of memory loss and neurobehavioral dysfunction (in vivo), promotion of activation of the sequestosome 1 (p62)/Keap1/Nrf2 pathway by inducing autophagy, suppressing ubiquitination of Nrf2, enhancing Nrf2 translocation into nucleus, reducing NQO1, inhibiting production of superoxide in mitochondria of neurons, lessening neuronal mitochondrial injury and increasing the downstream antioxidant proteins expression levels such as HO‐1. In addition, luteolin reduced the production of superoxides in neuronal mitochondria in a vitro trial and lowered the neuronal mitochondrial injury (Tan et al. 2019). Luteolin as a potent antioxidant and anti‐inflammatory agent is found to have a role in many brain disorders including Alzheimer's disease and Parkinson's disease by modulating multiple cellular pathways (Goyal, Solanki, and Verma 2024).

### 3.9 Luteolin in Alzheimer's Disease Treatment

Alzheimer's disease (AD) is the major cause of memory loss around the globe. Many studies suggested that in the pathology of AD, neuroinflammation is involved (Dhapola et al. 2021). The Amyloid‐β peptides (Aβ) in different forms are accumulated in extracellular matrix of the brain and act as key modulator of AD (Uwishema et al. 2022). It was found that fibrillary Aβ (fAβ) triggers and promotes inflammation by disrupting the blood–brain barrier (BBB). In vitro BBB model was designed by coculturing human astrocytes (hAs) and human brain microvascular endothelial cells (hBMECs) damaged by fAβ_1–40_ to investigate impact of luteolin on these cells by analyzing cytokine production, barrier function, cellular toxicity, and intracellular signaling pathways related to inflammation. The result revealed that cellular viability of hAs and hBMECs is enhanced by luteolin in fAβ_1–40_‐damaged cells. The increase in cytoprotection was also observed in basolateral and apical sides of coculture against the damage fAβ_1–40_‐induced damages. After exposure to fAβ_1–40_, barrier function is protected by luteolin via lessening aggravated permeability and safeguarding transendothelial electrical resistance in human BBB model. Furthermore, reduction in cytokine production including IL‐1β, IL‐6, IL‐8, TNF‐α, and COX‐2 as well as fAβ_1–40_‐induced inflammatory mediators after luteolin administration were observed. The mechanism to protect BBB against injuries induced by fAβ_1–40_ is based on regulating inflammatory signal transduction, which involves blockage of NF‐κB p65 nuclear translocation, reduction in discharge of inflammatory cytokines, relief of inhibitory κB α (IκBα) degradation, downregulation in the levels of phosphorylated inhibitory κB kinase (phosphor‐IKK), and inhibition of p38 MAPK activation. In a nutshell, luteolin has profound role in protection of BBB by blocking molecular activities and pathways related to inflammation and designated as potential therapeutic agent to reduce accumulation of Aβ, an essential aspect in AD treatment (Zhang, Tan, et al. 2017).

In transgenic drosophila, luteolin reduced Aβ42 peptides accumulation, acetylcholinesterase (AchE) activity, oxidative stress, increased lifespan, and improved cognitive dysfunction in AD flies. The mechanism behind these beneficial aspects of luteolin may include inhibition AchE activity, inhibition of Aβ42 aggregation, and direct ROS scavenging thereby overcoming the cognitive impairments and oxidative stress. These results also indicated that luteolin has a pivotal role in slowing down the inception of AD‐like symptoms (Ali et al. 2019). The memory and spatial learning impairment induced by streptozotocin (STZ) are substantially ameliorated by luteolin. Significant reduction in the thickness of CA1 pyramidal layer was noted due to STZ and luteolin treatment eliminated the STZ‐induced inhibitory effects (Wang et al. 2016; Sawmiller et al. 2014). The ultramironized composite of luteolin and palmitoylethanolamide was found to possess neuroprotective activities in AD. The composite was administered via intraperitoneal route to vehicle‐MOG35‐55‐immunized mice. The composite dose dependently improves the clinical score by reducing proinflammatory proteins NLRP3, and transcriptional expression of acute‐phase protein IL‐1β, TNF‐α, SAA1, IFN‐γ, and CB2 receptors and TCR‐ζ, CD3‐γ, CD137, Fpr2, TLR2 chain (Contarini et al. 2019). It also lowers ROS production, enhances the cell viability, reverses the mitochondrial membrane potential dissipation, decreases the Aβ_1‐42_ secretion, downregulates the AβPP level, and increases the SOD activity in AD cell model (Liu et al. 2011). In 3XTg‐AD mice, luteolin decreases protein plaques and improves histomorphology of brain as it limits the ER stress and retards neuro‐inflammatory aggravation which is the main cause of memory and learning impairments in mice (Kou et al. 2021). Furthermore, luteolin treatment inhibited neuroinflammation (IL‐1β, IL‐6, TNF‐α, NO, COX‐2, and iNOS proteins), astrocyte hyperactivation as well as improved spatial learning and reduced memory deficits in triple‐transgenic mouse model of AD (Kou et al. 2021). In brain tissue, ER stress markers expression (such as IRE1α and GRP78) also decreased. In addition, luteolin in hippocampal tissue of rats also prevents from cognitive impairment, learning and memory impairment, reductions in choline acetyl transferase activity, antioxidant enzymes concentrations, Bcl‐2/Bax ratio, and also enhancement in MDA level (Yu et al. 2015). At the lesion site, infiltration of immune cells and stimulation of active astrocytes were done via spinal cord injury whereas administration of luteolin (1 mg/kg/day i.p) for three consecutive days in infected mice enhanced the numbers of both doublecortin‐immunoreactive cells and bromodeoxyuridine‐positive nuclei as well as enhanced the expression of the nerve growth factor, brain‐derived neurotrophic factor, glial cell‐derived neurotrophic factor, and neurotrophin‐3 (Crupi et al. 2016).

Luteolin significantly stimulated the expression of Bcl2 and decreased the expression of caspase‐3 and BAX. In high concentration, luteolin inhibits Aβ25‐35 and induces cellular apoptosis. Luteolin activates the signaling pathways (ER/ERK/MAPK) and provide protection to Bcl2 cells from Aβ25‐35 and produce apoptotic bodies by direct stimulation of ERβ (Wang et al. 2020).

In a study, it was reported that luteolin have a role in reduction of apoptotic cell death, neuroinflammation, oxidative stress, synaptic dysfunction, and amyloid production via inhibition of JNK in Aβ1–42‐injected mice (Ahmad et al. 2021). In AD, glutamate accumulation is a key factor in neuronal cell death and luteolin has a potential role to activate mTORC1 and attenuate glutamate‐induced autophagy‐mediated cell death (Vongthip et al. 2024). Recently, neuroprotective role of luteolin during AD was found to be due to its role to bind directly with PPAR‐γ to enhance its expression and functional aspects which resultantly repair mitochondrial damages, reduce neuronal apoptosis, and inhibit generation of Aβ (He et al. 2023).

### 3.10 Parkinson's Disease Treatment and Role of Luteolin

Parkinson's disease (PD) is a progressive neurodegenerative illness that cause severe movement disorder and is manifested by bradykinesia and tremors (Raza and Anjum 2019; Hayes 2019). Luteolin neuroprotective activity is due to its potent antioxidative and anti‐inflammatory activity as well as its influence on multilayer modulatory pathways thus protecting against AD and PD (Goyal, Solanki, and Verma 2024). Due to oxidation stress, p35 and CDK‐5 expressions were noted to be remarkably high which are effectively suppressed by luteolin. This proves a luteolin dynamic role in influencing the Erk1/2‐ and Drp1‐dependent survival pathways (Reudhabibadh et al. 2021). In addition, luteolin reduced oxidative stress and apoptosis, mitochondrial damage and ROS production generated by 1‐methyl‐4‐phenylpyridinium iodide (MPP+) in neuroblastoma cells SH‐SY5Y (Reudhabibadh et al. 2021). In another study, luteolin‐7‐O‐glucoside was found to have a potent role in protection from dopaminergic neural injuries in the dopaminergic neural cell line SH‐SY5Y, where it enhanced the cell viability SH‐SY5Y cell line treated with MPP+ by decreasing nuclear condensation and suppressing apoptosis (Qin et al. 2019). Furthermore, it increases the neuro fibers in the striatum, restores the tyrosine hydroxylase (TH) positive neurons in the substantia nigra, reduces caspase‐3 to increase the Bcl2/BAX ratio, and consequently improves the performance of mice in behavioral tests, thereby establishing possibility of this molecule to be used in therapy of PD (Qin et al. 2019). Luteolin modulates PD associated genes, oxidative stress responses, and cellular pathways related to inflammation in microglial cell line BV2 and further providing the protection to these cells from rotenone toxicity (Elmazoglu et al. 2020). In addition, luteolin and palmitoylethanolamide ultramironized composite affect the camptothecin in PD patients. In PC12 cells, 6‐hydroxydopamine (6‐OHDA)‐induced apoptosis may result in loss of cell viability, increase in BAX/Bcl2 ratio and p53 expression. The luteolin treatment results in inhibition of apoptosis induced by 6‐OHDA and blocks mRNA expression of TRB3 and BIM, thus improving cell viability. This implicates neuroprotective activity of luteolin (Hu et al. 2014; Guo et al. 2012). In a recent research, endoplasmic reticulum (ER) stress was observed to the main cause of PD and ubiquitin ligase 3‐hydroxy‐3‐methylglutaryl‐coenzyme A reductase degradation 1 (HRD1) and its stabilizing molecule, the suppressor/enhancer lin‐12‐like (SEL1L) have a profound role in suppression ER stress Hence, luteolin suppress ER stress and activates HRD1 and SEL1L and might be a drug of choice to treat PD (Nishiguchi et al. 2024). More extensive research is still needed to ascertain the role of luteolin as a therapeutic drug against PD (Brenot et al. 2018).

### 3.11 Antiasthmatic Role of Luteolin

Asthma is a chronic disease of the lungs and is characterized by airway smooth muscle hypertrophy, epithelial shedding, airway obstruction due to inflammation, hyperresponsiveness of bronchi due to overproduction of airway mucus or narrowing of bronchial tubes. The clinical symptoms include coughing, wheezing when exhaling, shortness of breath, and chest pain or tightness with recurrent episodes (Gans and Gavrilova 2020; Sockrider and Fussner 2020). Ovalbumin (OV) is used to induce asthma in a vivo model of mice whereas supplementation of luteolin in lung tissues at the rate of 10 mg/kg has been found to lower goblet cells number and limit the mucus overproduction as well as suppress the GABA_A_ receptors (GABA_A_R)‐mediated currents in A549 cells (Shen et al. 2016). Moreover, in a trial using BALB/c mice sensitized by OV antigen, it was observed that luteolin could effectively impede airways inflammation and upregulation of T helper 2 (Th2) because this molecule substantially decreased the immunoglobulin E (IgE), IL‐13, IL‐5, and IL‐4 levels in bronchoalveolar lavage fluid (BALF), as well as CD4 + T, CD3‐CCR3+, CD3e+Gr‐1+ and CD19+B cells, in pulmonary tissues whereas Th1 cytokine, that is, interferon‐ γ (IFN‐γ) was raised after treatment with luteolin. In a in vitro trial, it was found that CD4+foxp3+CD25+inducible regulatory T (iTreg) cells are involved in amplification of functional activity of Treg cells. The mRNA expression levels of transforming growth factor‐β1 (TGF‐ β1) and foxp3 were raised in lung tissues after induction of adoptively transfer of these cells by luteolin. In an allergen inhalation experiment, iTreg cells were introduced into OVA‐sensitized mice which resulted in reduction of IgE, eotaxin, and Th2 cytokine expression, hypersensitivity of airways, recruitment of eosinophil, as well as increased in the production of IFN‐γ in BALF. Moreover, in an asthma mouse model, adoptive transfer of iTreg cells results in protection from illness due to reduction of CD25. In addition, luteolin activates Treg cells that results in decrease of inflammation and increase in autoimmunity during allergic asthma (Seumois et al. 2020; Lan et al. 2020). Luteolin stimulates CD4+CD25+Treg cells and foxp3 which is an effective approach in the management of asthma (Kim et al. 2018). In another previous study, intraperitoneal administration of luteolin (0.1 mg/kg) to BALB/c mice in BALF exhibited multiple changes such as reduction in the number of neutrophils, eosinophils, expressions of IL‐4, IL‐5 and IL‐13, and lymphocytes, along with decrease in inflammatory cell infiltration (Jang et al. 2017).

In another study on allergic rhinitis in house dust mite (HDM)‐sensitized BALB/c mice reported reduction in CD4+IL‐4‐secreting, serum HDM‐specific IgE, T cells, decrease in nasal epithelial secretion of mucus, infiltration of eosinophils expression of pSTAT6 and GATA3, decline in percentage of CD4+IL‐4‐secreting splenocytes expression and reduction in allergic symptoms after luteolin treatment (Liang et al. 2020). The results of a recent study on human ANO1‐HEK293T cells revealed that luteolin significantly constrained human ANO1 activity. Further, luteolin and *Spirodela polyrhiza* ethanolic extract was found to have no effect on cystic fibrosis transmembrane conductance regulator (CFTR) current and explicitly controlled the calcium‐activated chloride current in cultured IL‐4 sensitized Calu‐3 cells of human airway epithelium. Similarly, in Calu‐3 cells after sensitization by IL‐4, the ATP‐induced increase in electrolyte transport was significantly inhibited by luteolin (Kim et al. 2020). In a recent study, it was noted that autophagy in the pulmonary tissues was inhibited in asthmatic mice by luteolin via restricting beclin 1‐PI3KC3 protein complex and activating PI3K/Akt/mTOR signaling pathway (Wang, Zeng, et al. 2021).

The inflammation in bronchial tubules of lungs worsens the asthma which is also associated with Th2 cytokines release such as IL‐4 and IL‐13, that resultantly involve in remodeling of markers related to M2 macrophages (Shen et al. 2018; Brenot et al. 2018). To treat asthma, reduction in M1 macrophages which are abundantly present in asthmatic patients and increase in M2 macrophages is crucial to overcome asthmatic conditions (Lee, Park, Bae, et al. 2021). Recently, it was observed that luteolin reduced M1 polarization and enhanced M2 polarization of macrophages by stimulation of THP‐1. The USP4 and hsa‐miR‐136‐5p expressions were also effectively mediated by luteolin and found to be a vital approach in treatment of asthma (Gong et al. 2022). Recently, it was observed that luteolin alleviates the inflammatory responses and secretions of inflammatory factors in human bronchial epithelial cells during asthma by inhibiting IL‐1β and MAPK signaling and reduced the secretion of IL‐36γ (Qiao et al. 2023).

### 3.12 Toxicological Profiles of Luteolin

Despite numerous pharmacological activities related to prevention from cancer, cardiovascular disorders, diabetes, obesity, neurodegenerative, and other diseases, the safety profile of luteolin needs further exploration. Prior to harnessing the potent role in inflammatory and many other diseases, toxicological considerations of luteolin are crucial to overcome potential adverse effects. The results of several studies elucidated luteolin as a novel drug impacting transcription factors, protein expressions and modulation of cellular pathways related to disease prevention but the information regarding its safe use and toxicity profile to ascertain its therapeutic benefits effectively for clinical translation is still needed. A precise dose of luteolin against particular disease is crucial as its administration may affect many other cellular structures or pathways. A study to determine toxicological profile of luteolin was conducted using human retinal microvascular endothelial cells and noted that low dose (10 μM) imparts no toxic effect but affected the cells with high dose (100 μM) (Caporali et al. 2022; Park et al. 2012). The 100 μg/mL administration of luteolin was found to cause DNA damages in lymphocytes and Vero cells (Cariddi et al. 2015). In another experiment on female Sprague–Dawley rats, prenatal toxicity was noted when a medicinal herb ( *Verbena officinalis* ) containing luteolin was given in high doses during the gestation period (Fateh et al. 2019). Likewise, human lymphoblastoid TK6 cells showed cytotoxic activity after 24 h of luteolin treatment and 2.5 μM of luteolin was found to be a minimal lethal dose concentration whereas luteolin concentration of 5 and 10 μM results in DNA damage (Li et al. 2021).

In an experiment, luteolin was orally given in doses of 50, 100, and 200 mg/kg to determine its impact on blood and liver function tests. The results indicated elevated levels of RBCs, PCV, and Hb but WBCs remains unaffected. A significant dose dependent increase in liver enzymes was observed including alkaline phosphatase, aspartate transaminase, and alanine transaminase. The histological data reveals no damage to liver (Orji et al. 2020). Doxorubicin, a drug used in treatment of lung cancer, has various adverse outcomes. Luteolin cotreatment reduces these negative impacts such as reduced the production of apoptotic indicators and proinflammatory cytokines; elevated the number of platelets, RBCs, WBCs, and minimized the pathological damages to the lungs (Owumi et al. 2021). As high doses of luteolin have negative consequences, hence optimization of standard doses is required for its wider therapeutic benefits and effective clinical applications.

## 4 Conclusion

The luteolin has wide distribution in many plant families and more than 300 species were documented to possess luteolin and its glycosides. It has been used in various traditional medicine systems to treat various ailments. The bioavailability of luteolin is very low although it is abundantly present in dietary sources which limit its clinical applications. To improve bioavailability, excessive glucuronidation should be controlled using potent inhibitors like resveratrol. In addition, solubility, permeability, and photoprotective activity of luteolin can be improved by various methods such as glycosylation, nanoparticles encapsulation, and microemulsion system. This suggests that luteolin's bioavailability can further be increased by using other modern procedures. Luteolin was found to be a potent phytochemical against various diseases including cancer, cardiovascular disorders, diabetes, obesity, inflammation, hepatic disorders, renal disorders, brain injury, AD, PD, and asthma. Luteolin is effectively involved in the regulation of various mediators related to signaling and inflammatory pathways and can be helpful in treating various chronic diseases. More extensive research at molecular level should be carried out to ascertain the exact mechanisms behind beneficial activity of luteolin to overcome diseases.

## Author Contributions

**Mahwish:** writing – original draft (equal). **Muhammad Imran:** conceptualization (equal), writing – original draft (equal). **Hammad Naeem:** methodology (equal), writing – original draft (equal). **Muzzamal Hussain:** investigation (equal), writing – original draft (equal). **Suliman A. Alsagaby:** data curation (equal), visualization (equal). **Waleed Al Abdulmonem:** resources (equal), validation (equal), visualization (equal). **Ahmed Mujtaba:** resources (equal), software (equal). **Mohamed A. Abdelgawad:** writing – review and editing (equal). **Mohammed M. Ghoneim:** writing – review and editing (equal). **Ahmed H. El‐Ghorab:** writing – review and editing (equal). **Samy Selim:** data curation (equal), resources (equal). **Soad K. Al Jaouni:** resources (equal), supervision (equal). **Ehab M. Mostafa:** data curation (equal), investigation (equal), project administration (equal). **Tadesse Fenta Yehuala:** supervision (equal), writing – review and editing (equal).

## Conflicts of Interest

The authors declare no conflicts of interest.

## Data Availability

The data that support the findings of this study are available within the article.
